# Entomological surveillance with viral tracking demonstrates a migrated viral strain caused dengue epidemic in July, 2017 in Sri Lanka

**DOI:** 10.1371/journal.pone.0231408

**Published:** 2020-05-06

**Authors:** Gayan P. Withanage, Hapuarachchige C. Hapuarachchi, Sameera D. Viswakula, Y. I. Nilmini Silva Gunawardena, Menaka Hapugoda

**Affiliations:** 1 Molecular Medicine Unit, Faculty of Medicine, University of Kelaniya, Ragama, Sri Lanka; 2 Environmental Health Institute, National Environment Agency, Singapore, Singapore; 3 Department of Statistics, Faculty of Science, University of Colombo, Colombo, Sri Lanka; Faculty of Science, Ain Shams University (ASU), EGYPT

## Abstract

Dengue is the most important mosquito-borne viral infection disease in Sri Lanka triggering extensive economic and social burden in the country. Even after numerous source reduction programmes, more than 30,000 incidences are reporting in the country every year. The last and greatest dengue epidemic in the country was reported in July, 2017 with more than 300 dengue related deaths and the highest number of dengue incidences were reported from the District of Gampaha. There is no Dengue Virus (DENV) detection system in field specimens in the district yet and therefore the aim of the study is development of entomological surveillance approach through vector survey programmes together with molecular and phylogenetic methods to identify detection of DENV serotypes circulation in order to minimize adverse effects of imminent dengue outbreaks. Entomological surveys were conducted in five study areas in the district for 36 months and altogether, 10,616 potential breeding places were investigated and 423 were positive for immature stages of dengue vector mosquitoes. During adult collections, 2,718 dengue vector mosquitoes were collected and 4.6% (n = 124) were *Aedes aegypti*. While entomological indices demonstrate various correlations with meteorological variables and reported dengue incidences, the mosquito pools collected during the epidemic in 2017 were positive for DENV. The results of the phylogenetic analysis illustrated that Envelope (E) gene sequences derived from the isolated DENV belongs to the Clade Ib of Cosmopolitan genotype of the DENV serotype 2 which has been the dominant stain in South-East Asian evidencing that a recent migration of DENV strain to Sri Lanka.

## Introduction

Dengue is an arthropod-borne viral infection found the throughout tropical and subtropical regions of the world. Over the past 50 years, dengue has become the most rapidly spreading disease in the world with a 30-fold magnitude rise of global incidences. According to the World Health Organization (WHO), more than 50 billion people have been infected with dengue around the globe with 50–100 million annual incidences [1]. The causative agent of the disease is one of the four antigenically distinct serotypes Dengue Virus (DENV) which is a positive stranded RNA virus belonging to the genus Flavivirus of the family Flaviviridae. The disease is transmitted to humans mainly via bites of *Aedes (Stegomyia)* dengue vector mosquitoes. *Aedes aegypti* (Linnaeus) is considered as the predominant vector while *Ae*. *albopictus* (Skuse) is considered as the subsidiary vector of DENV. Transmission of dengue is mainly influenced by large-scale unplanned urbanization and the increase in human population. Further, increased global travelling has also been identified to be contributing significantly for the spread of the virus [2, 3].

The first dengue incidence in Sri Lanka was reported in 1962 and ever since, the number of dengue incidences increased every year. The first Dengue Hemorrhagic Fever (DHF) epidemic was reported in Sri Lanka in 1989 and after that DHF has been considered endemic to Sri Lanka [4]. All four serotypes of DENV are circulating in the country even though the predominant serotype is changing over time [5]. Sri Lanka experienced a recent dengue epidemic in 2017 with more than 186 000 dengue incidences with 215 deaths. Approximately half of the total dengue incidences were reported in the Western Province which comprises of Districts of Colombo, Gampaha and Kaluthara. The highest number of dengue incidences were reported in the District of Colombo which is the commercial capital and largest city of the country followed by the District of Gampaha bordering the Northern boundary of the District of Colombo. Despite many efforts taken for controlling dengue in the District of Gampaha, more than 5,000 cases on average are reported annually with approximately 32,000 reported cases in the year 2017 [6]. It is required to have a broad knowledge on entomological risk factors, distribution, densities and seasonal variation of dengue vector mosquitoes together with climatic and other possible risk factors affecting transmission of dengue to control dengue incidences in the District of Gampaha, however, there is no study conducted in the district to identify DENV serotypes in specimens from entomological surveys and identification of its origin and circulation. Therefore, the study is focused on development of better entomological surveillance approach through vector survey programmes together with molecular and phylogenetic methods to identify detection of DENV serotypes circulation in order to minimize adverse effects of imminent dengue outbreaks.

## Materials and methods

### Selection of study areas

**Fig 1:**
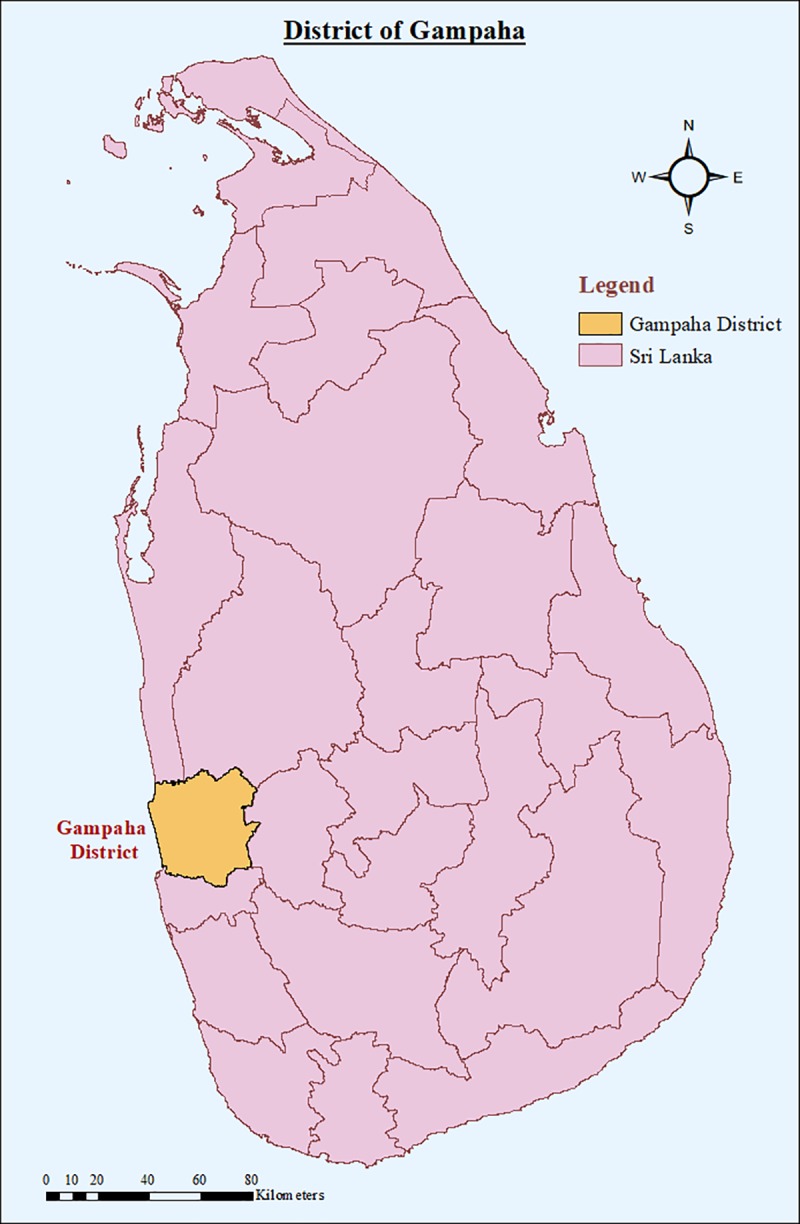
The district of Gampaha of Sri Lanka. The map was developed using the layers provided by the Survey Department, Sri Lanka and the figure was generated using ArcGIS software (version 10.2).

The District of Gampaha (Fig 1) where the second highest number of dengue cases reported in Sri Lanka [6] was selected for the study. District of Gampaha is located in the Western Province in the Wet Zone and expands over an area of 1,387 square (km^2^). Elevation of the district ranges from sea level to 450 m. The district experiences a temperature in the range of 21.6–37 ^o^C with average annual rainfall of 1,750 mm. Precipitations are mainly received during the Southwest monsoon and second inter-monsoon seasons [7].

District of Gampaha is the second most populated district with the third highest urban population in Sri Lanka with an estimated population of around 2.2 million. The district had an annual population growth rate of 1.02% in the year 2008. Depending on the presence of major cities and industrial sites the population density varies [8]. In 2004/2005, the area of paddy cultivated was 10,170 hectares in the district. Fifteen Medical Officer of Health (MOH) areas with 1,177 Grama Niladhari (GN) divisions and 1,784 villages are located in the district [9–11].

#### Permission from the ethical review committee and health authorities

Ethical permission for the study was obtained from the Ethical Review Committee of the Faculty of Medicine, University of Kelaniya, Sri Lanka (Ref. No. P/238/12/2014). Permission to conduct the study was obtained from the Regional Director of Health Services and Regional Epidemiologist of the District of Gampaha. The MOH officers and Public Health Inspectors (PHI) in study areas were informed prior to initiating the study.

### Monitoring of entomological risk factors

#### Selecting sampling sites

Number of dengue incidences reported in past ten years (2005–2014) in the District of Gampaha were collected from all the MOH officers and Regional Epidemiologist, Gampaha. High (n = 4) and low (n = 1) dengue transmission areas were identified based on mean reported dengue incidences in each MOH area. Four MOH areas with an average number of dengue incidences greater than 300 per year were selected as dengue high risk areas. When selecting sampling sites, one GN division reporting the highest number of dengue incidences during the period of 2012–2014 in each selected MOH area was selected. One MOH area with the lowest number of dengue incidences was selected as dengue low risk area. One GN division reporting the lowest dengue incidences during the same period of 2012–2014 was selected when selecting the sampling site in the selected control MOH area.

In this study, number of breeding places per household was considered as the primary response. Since it is a count, the following sample size calculation formula was used assuming a Poisson distribution [12].

$$n\approx {\left(\frac{200}{r}\right)}^{2}.\frac{1}{\bar{x}}$$

where,

n = Sample size required for a Poisson variable

r = Desired relative error (as percentage)

$\frac{1}{\sqrt{\bar{x}}}$ = Coefficient of variation

Following the calculation, *n* was equal to 128 with a Poisson mean of 2 and 25% precision. Then, with a 15–20% non-responsive rate, a sample size was considered as 150 households in the current study.

A simple random sampling approach was used to select 150 households in each selected GN division. Each household was labelled with a unique identification number. In addition, abandoned houses, dumping yards and religious places in the study areas were considered as open areas. They also were marked with an identification number. Community awareness was performed by visiting to each household in each study/control site prior to initiate the study.

#### Entomological surveillance

Monthly entomological surveillance was conducted in the study sites for three years (June, 2015—May, 2018). All the findings of monthly entomological surveillance data were entered into a specially designed form.

*Collection of adult dengue vector mosquitoes*. Adult dengue vector mosquitoes were collected mainly using mouth aspirators. In addition, back-pack aspirators and hand-catching nets were also used to collect adult mosquitoes. Both indoor and outdoor resting and landing dengue vector mosquitoes were collected following WHO guidelines [13]. The collections were initiated in the dawn around 6.00 Ante Meridiem (a.m.) in the study households and continued till around 11.00 a.m. While every outdoor potential resting places were examined, bed rooms, coat racks, curtains, indoor flower trees and potential dark places were examined in the household interior. In each household, adult collections were conducted for 15 minutes. Special precautions were taken to avoid risk of accidental biting by mosquitoes.

*Identification of breeding containers and collection of immature stages of dengue vector mosquito samples*. All potential breeding places in each premise were examined while wet and positive containers were recorded separately. Positive containers were divided into three groups depending on the mosquito species present *viz*. *Ae*. *aegypti*, *Ae albopictus* and other species. Larval and pupal specimens were collected carefully using a glass pipette and transferred to labelled vials with the house-hold number, locality, date of collection and type of breeding place. These breeding places were categorized as natural or man-made containers and a directory was prepared for dengue vector breeding sites including each breeding place. The directory was updated each month as new breeding places were examined.

*Transferring*, *identification and storage of mosquito samples*. The field-caught adult mosquitoes were transferred into vials or paper cups covered with mosquito nettings and labelled with locality, household or open area number, date of collection and the genera of the mosquitoes accordingly. Further, collected immature stages of dengue vector mosquitoes from positive containers in the study areas were transferred to separate vials and labelled accordingly as mentioned previously. Field collected specimens were identified using standard mosquito keys [14–16] under a light microscope prior to store at -80°C until tested by molecular-based assays.

*Investigation of distribution of studied variables in study and control areas*. Studied variables during the entomological survey, namely number of reported dengue incidences, total mosquito count, filed collected dengue vector mosquito counts in each species, and dry, wet and positive containers in study and control areas were analyzed using Kruskal-Wallis test with post-hoc analysis.

*Investigation of correlations between entomological findings with climatic variables*. Pearson correlation analysis was used to identify the associations among the meteorological variables, namely rainfall, number of rainy days, minimum and maximum temperature, minimum and maximum Relative Humidity (RH), average wind speed, with monthly captured *Aedes* adult dengue vector mosquitoes initially. Then, associations of above environmental variables with entomological variables, namely monthly observed dry, wet, total positive containers for immature stages of dengue vector mosquitoes and containers positive for *Ae*. *aegypti* and *Ae*. *albopictus* respectively were studied with three month lag period. Significant associations were further examined using multivariate regression model to determine the best predictor variables associated with relative abundance of the larval and adult densities.

*Identification of correlations between entomological findings with dengue incidences*. Different entomological indices such as House index (HI), Container index (CI) and Breteau index (BI) [17] were calculated using formulas given in S1 File. Pearson correlation analysis was used to investigate relationships and correlations among studied entomological variables, namely monthly observed dry, wet, total positive containers for immature stages of dengue vector mosquitoes and containers positive for *Ae*. *aegypti* and *Ae*. *albopictus* respectively together with the results of mosquito indices (HI, CI and BI) with reported dengue incidences in each study area.

#### Identification of DENV transmitting by field-caught mosquitoes

Real-Time Reverse Transcriptase Polymerase Chain Reaction (rRT-PCR) methodology was developed primers proposed by Lanciotti *et al*. [18] for detection of DENV and serotyping of DENV were established.

Oligonucleotide primers (S2 File, Table A1) designed between C and PrM regions of the DENV genome by Lanciotti *et al*. (1992) purchased from Integrated DNA Technologies, USA were used for detection and serotyping of DENV.

*Ae*. *albopictus* C6/36 cloned cell lines infected separately with reference strains of DENV1-4 [19] were kindly provided by the Genetech, Sri Lanka. RNA was extracted from field collected specimens following methods described by Boom *et al*. [20] with some modifications. Briefly, an aliquot of 140 μl of specimen samples were transferred to 1.5 ml micro-centrifuge tube containing 10 μl of acid treated Silica Coarse (SC) and 1 ml of lysis buffer. Then, the solution was vortexed for 15 seconds followed by incubation for 5 minutes at room temperature. After that, the tube was vortexed again for 15 seconds and incubated for 5 minutes at room temperature. Then, the tube was centrifuged at 12,000 rpm for 10 seconds at room temperature and the supernatant was discarded. Next, an aliquot of 1 ml of washing buffer was transferred into the micro-centrifuge tube and the solution was mixed well by vortexing for 15 seconds. Then, the tube was centrifuged at 12,000 rpm for 10 seconds at room temperature and the supernatant was discarded. The washing step was repeated again. An aliquot of 500 μl of double DW was added and the tube was inverted until the silica pellet was dissolved. After centrifuging at 12,000 rpm for 10 seconds, the supernatant was discarded. Next, the tube was centrifuged again at 12,000 rpm for 10 seconds again and the remaining water was removed carefully. Then, an aliquot of 20 μl of PCR water was added and the SC pellet was dissolved well by tapping prior to incubation at 56°C for 10 minutes. After the incubation period, the tube was centrifuged at 12,000 rpm for 10 seconds and an aliquot of 14 μl of the supernatant was transferred into a 1.5 ml new micro-centrifuge tube. An aliquot of 4U of RNase inhibitor (RNaseOut^TM^, Invitrogen, USA) was added before storing the isolated RNA at -20°C (R600a, HISENSE, China).

*Testing field-caught dengue vector mosquito samples by molecular-based assays to detect DENV transmission*. Field-caught dengue vector mosquito specimens (eggs, larvae and adults) were subjected to molecular experiments to identify transmission of DENV in the field.

*Extraction of dengue viral RNA*. Adult mosquitoes—Field-caught adult dengue vector mosquitoes were pooled together depending on the species, collection date and study area and each pool included maximum of 50 mosquitoes. In each pool, head and thorax of each mosquito was separated after removing abdomen, legs and wings. Then, an aliquot of 160 μl of autoclaved Phosphate Buffered Saline (PBS) was added to the pool and crushed well using autoclaved plastic rods. After crushing, the samples were centrifuged at 4,000 rpm for 1 minute time period and an aliquot of 140 μl of the supernatant was transferred to another 1.5 ml micro-centrifuge tube containing 10 μl of SC and 1 ml of lysis buffer. Then, the RNA extraction was performed as mentioned above.

Eggs—Field collected eggs of dengue vector mosquitoes were pooled together and directly subjected to RNA extractions. An aliquot of 160 μl of autoclaved PBS was added to egg pool and crushed well using autoclaved plastic rods. After crushing, the samples were centrifuged at 4,000 rpm for 1 minute time period and an aliquot of 140 μl of the supernatant was transferred to another 1.5 ml micro-centrifuge tube containing 10 μl of SC and lysis buffer. The RNA extraction was performed as mentioned above.

Larvae—Field collected larval stages of dengue vector mosquitoes were pooled together each pool was transferred to separate 1.5 ml micro-centrifuge tubes containing 160 μl of PBS carefully and the larvae were crushed well using autoclaved plastic rods. After crushing the samples, rest of the extraction was performed as mentioned above.

*Reverse-transcription of RNA*. Extracted RNA from adult female dengue vector mosquitoes, eggs and larvae were subjected to reverse transcription. Moloney Murine Leukemia Virus (M-MLV) Reverse Transcriptase (Invitrogen, USA) was used to prepare cDNA. For the reverse transcription and PCR assay optimizations, Deoxynucleotide triphosphate (dNTP) solutions were purchased from Promega, USA. Mixture 1 was prepared using 3.0 μl of RNA and 0.4 μM of D2 reverse primer and the total volume of Mixture 1 was adjusted to 12 μl using PCR water. The Mixture 2 was consist of 1X First-Strand RT enzyme Buffer, 2.0 mM of each dNTP, 4 U of RNase inhibitor, 200 U of M-MLV RT enzyme and the total volume of Mixture 2 was adjusted to 13 μl using PCR water. Upon preparation of Mixture 1, the PCR tube was incubated at 65°C for 5 minutes followed by keeping 3 minutes in ice prior to addition of ice cold Mixture 2. After the addition of Mixture 2, the tube was incubated at 37°C for 60 minutes and prepared cDNA was stored at -20°C until tested by PCR.

*Detection of DENV and serotypes*. Prepared cDNA was then used for rRT-PCR to identify the presence of DENV in field-collected specimens. For the first PCR of detection of DENV, assay conditions were used following manufacturer’s guidelines of QuantiTect^®^ SYBR^®^ Green PCR kit (Qiagen-USA) with 5 μl of cDNA. Thermal cycling profile was as follows: an initial denaturation at 94°C for 10 minutes, 35 cycles of denaturation at 94°C for 30 seconds, annealing at 54°C for 60 seconds, extension at 72°C for 60 seconds followed by a final extension at 72°C for 7 minutes. Melting analysis was then performed starting from 65°C with gradually increment of temperature by 0.5°C at each step of 20 seconds until 95°C. The rRT-PCR was performed using the same Real Time Thermal Cycler (ESCO, USA).

Five times diluted products of the first PCR were used as the template for the semi-nested PCR to identify the DENV serotypes. Here, assay conditions were used following manufacturer’s guidelines of same RT-PCR kit with the four serotype specific reverse primers. An aliquot of 3 μl of diluted PCR product was used as the template for the serotyping assay. Thermal cycling profile was as follows: an initial denaturation at 94°C for 10 minutes, 35 cycles of denaturation at 94°C for 30 seconds, annealing at 54°C for 60 seconds, extension at 72°C for 60 seconds followed by a final extension at 72°C for 7 minutes. Melting analysis was performed starting from 65°C with gradually increment of temperature by 0.1°C at each step of 20 seconds until 95°C.

*Genotyping of DENV*. After detection of serotype of DENV in positive pools, another PCR was performed to amplify complete E gene with serotype specific primers with the objective of identification of the genotype of the DENV. The extracted RNA were reverse transcribed using random oligonucleotide hexamers (Promega Corp., USA) as mentioned previously.

The PCR was performed using following assay conditions: 1X colorless Go*Taq*^TM^ reaction buffer, 1.5 mM of MgCl_2_, 0.8 mM of dNTPs, 0.2 μM of each primer and 2.5 U of Go*Taq*^®^ DNA polymerase. An aliquot of 5 μl of prepared cDNA was used for the assay and the total volume of the reaction mixture was adjusted to 50 μl using PCR water. The Den2_771F and Den2_2540R DENV serotype 2 specific primers (S2 File, Table A2) were used to amplify complete E gene. Thermal cycling profile was as follows: an initial denaturation at 94°C for 10 minutes, 40 cycles of denaturation at 94°C for 30 seconds, annealing at 58°C for 90 seconds, extension at 72°C for 2 minutes followed by a final extension at 72°C for 7 minutes. The amplified PCR products were size fractionated using agarose gel electrophoresis as mentioned in Chapter 3.3.1.4.e.

Upon completion of the genotyping PCR, the PCR products were purified using the GeneJET PCR purification kit (Thermo Fisher Scientific, USA) following manufacturer’s instructions, before being sequenced at a commercial facility using BigDye Terminator v3.1 cycle sequencing kit (Thermo Fisher Scientific, USA).

*Generation of sequences and multiple sequences alignment*. The forward and reverse chromatograms were assembled in Lasergene 8.00 software suite (DNASTAR Inc., USA). For multiple sequence alignment, complete E gene sequences of DENV that are reported from different geographic locations were downloaded from the GenBank database (S3 File) and the consensus sequences were aligned using MAFFT version 7 [21–23].

*Phylogenetic analysis of generated sequences*. Phylogenetic tree was developed using MEGA7 software with General Time Reversible (GTR) substitution model with gamma distributed rates. The robustness of clades was determined by using bootstrap analysis of 500 replicates.

*Development of evolutionary network of DENV*. Evolutionary network was constructed using the Network (version 5.0.0.3) software to visualize the complex evolutionary processes of DENV E gene sequences. After transforming the aligned sequences to Phylip format (phy 3.2) using the BioEdit (version 7.2.5), the alignment was feed to Network software (version 5.0.0.3) to construct network with Median-Joining algorithm.

*Phylogeography analysis of spatio-temporal spread of DENV*. The Bayesian Evolutionary Analysis by Sampling Trees (BEAST) software package was used to analyze spatio-temporal spread of DENV using Bayesian Markov chain Monte Carlo (MCMC) statistical framework. Sequences in the alignment was renamed in the format of Accession No._Country_Year prior to transforming the alignment to nexus (.nex) format. The nexus file was feed to the Bayesian Evolutionary Analysis Utility (BEAUti) and the clock model parameter was changed to “New trait” partition. In the ‘Tips’ tab, ‘Use tip dates’ was checked and ‘Chain Length’ was set to 10 000 000 iterations in the ‘MCMC’ tab. The edits were saved to.xml file which was feed to BEAST. Upon completion of BEAST run, the ESS values were examined using the trace log in Tracer software. The Maximum Clade Credibility (MCC) tree was developed using the Tree Annotator software using the “Location Tree” log file. Discrete phylogenetic module was utilized for the visualization of the generated MCC tree [24]. The output tree file of the BEAST run was feed to SPREAD (version 1.0.7) [25] and the location coordinates were fed using the ‘Setup’ tab. The “Most recent sampling date” is set to 20th June, 2017 prior to generating the.kml file. The generated.kml file was visualized using the Google Earth Pro (version 7.3.2.5491-64-bit) software.

## Results

**Fig 2:**
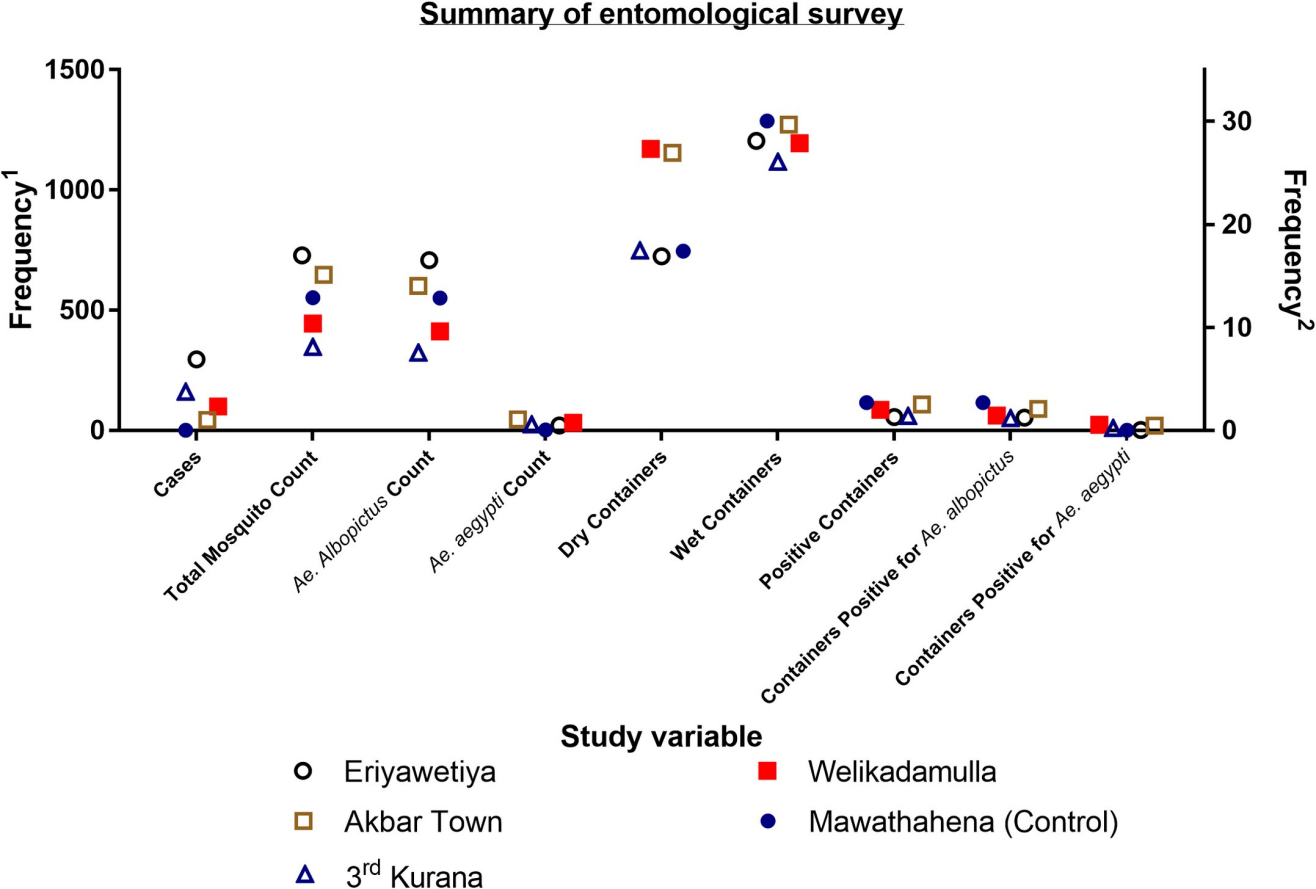
Summary of the entomological survey.

During the entomological survey, a total of 10,616 potential breeding containers were observed from all study areas and 2,718 dengue vector mosquitoes were collected and 4.56% of them were *Ae*. *aegypti* mosquitoes. The results of the entomological survey were summarized in the Table 1 and Fig 2. The highest number of mosquitoes (n = 728) were captured from the Eriyawetiya dengue high risk study area and highest number of potential containers (n = 2,426) were observed from the Akbar Town high risk area from which 52.4% (n = 1,272) were wet containers. In the high risk areas, the highest percentage of wet containers were reported from the Eriyawetiya study area (62.45%, n = 1,204) and the lowest percentage was reported from the Welikadamulla (50.5%, n = 1,194) study area. The highest percentage of positive containers for immature stages of dengue vector mosquitoes in the high risk areas was reported from the Akbar Town study area (8.5%, n = 108) from which 17.6% was positive for *Ae*. *aegypti* (n = 19). When considering the whole study population, the highest percentage of wet containers and positive containers for immature stages of dengue vector mosquitoes were reported from the control area (63.3% and 9.0% respectively). All the breeding places of immature stages of dengue vector mosquitoes in the control area were positive for *Ae*. *albopictus*. However, no significant difference was observed in the distributions of total mosquito count and counts of dry, wet and total positive containers in the high risk study areas compared to the control area according to the Kruskal-Wallis test with Bonferroni post-hoc analysis. When comparing the number of field caught adult *Ae*. *aegypti* mosquitoes in the study populations using Kruskal-Wallis test, significant difference was observed in high risk areas compared to the control area (H(4) = 17.97, *p* = 0.00125) and it was significantly higher in Eriyawetiya (*p* = 0.0268), Akbar Town ((*p* = 0.0007) and 3^rd^ Kurana (*p* = 0.0221) study areas. Further, significant difference was observed when analyzing the containers that were positive for immature stages of dengue vector mosquitoes. When comparing to the control, the number of breeding places of *Ae*. *albopictus* was significant differed in high risk areas (H(4) = 11.87, *p* = 0.0184) and it was significantly lower in 3^rd^ Kurana (*p* = 0.0256) study area.

**Table 1:** Summary of the entomology survey conducted during June, 2015 to May, 2018.

Area	*Ae*. *albopictus* count	*Ae*. *aegypti* count	Dry containers	Wet containers (%^1^)	Positive wet containers (%^2^)	Total containers positive for *Ae*. *albopictus* (%^3^)	Total containers positive for *Ae*. *aegypti* (%^4^)
Eriyawetiya	708	20	724	1204 (62.45)	55 (4.57)	53 (96.36)	2 (3.64)
Akbar town	601	46	1154	1272 (52.43)	108 (8.49)	89 (82.41)	19 (17.59)
3^rd^ Kurana	323	24	748	1117 (59.89)	59 (5.28)	50 (84.75)	9 (15.25)
Welikadamulla	412	32	1171	1194 (50.49)	85 (7.11)	62 (72.94)	23 (27.06)
Mawathahena (Control)	550	2	745	1287 (63.34)	116 (9.01)	116 (100.00)	0 (0.0)
Total	2594	124	4542	6074	423	370	53

^1^Percentage of wet containers from total containers,

^2^Percentage of positive containers from total wet containers

^3^Percentage of positive containers for *Ae*. *albopictus* from total positive containers

^4^Percentage of positive containers for *Ae*. *aegypti* from total positive containers

**Fig 3:**
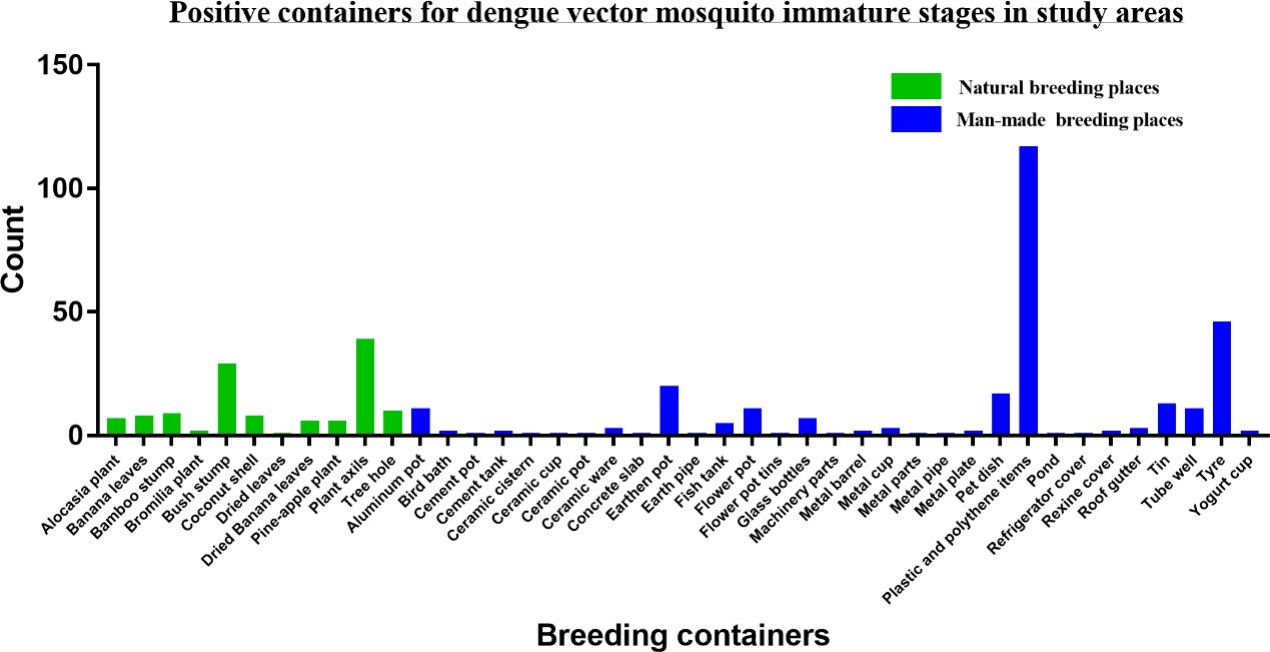
Summary of positive breeding containers of dengue vector mosquitoes in the study areas.

The observed positive breeding places were categorized into two groups namely, man-made (artificial) and natural breeding places. The summary of the two categories were shown in Fig 3. A total of 416 breeding containers were positive for immature stages of dengue vector mosquitoes from which 70.0% (n = 291) were man-made containers and 30% (n = 125) were natural breeding places in all study areas. The most prominent man-made positive containers for dengue mosquito life stages was plastic and polythene containers (28.1%, n = 117) and the main natural breeding places were plant axils (9.4%, n = 39). Frequently observed other man-made breeding places were discarded tires (11.1%, n = 46), earthen pot (4.8%, n = 20), pet dish (4.1%, n = 17) and discarded tins (3.1%, n = 13). The highlighted other natural breeding places were bush-stumps (6.9%, n = 29) and tree holes (2.4%, n = 10). Summary of the categories of positive breeding places was show in Table A3 in S2 File.

### Variations of entomological indices in the study areas

**Fig 4:**
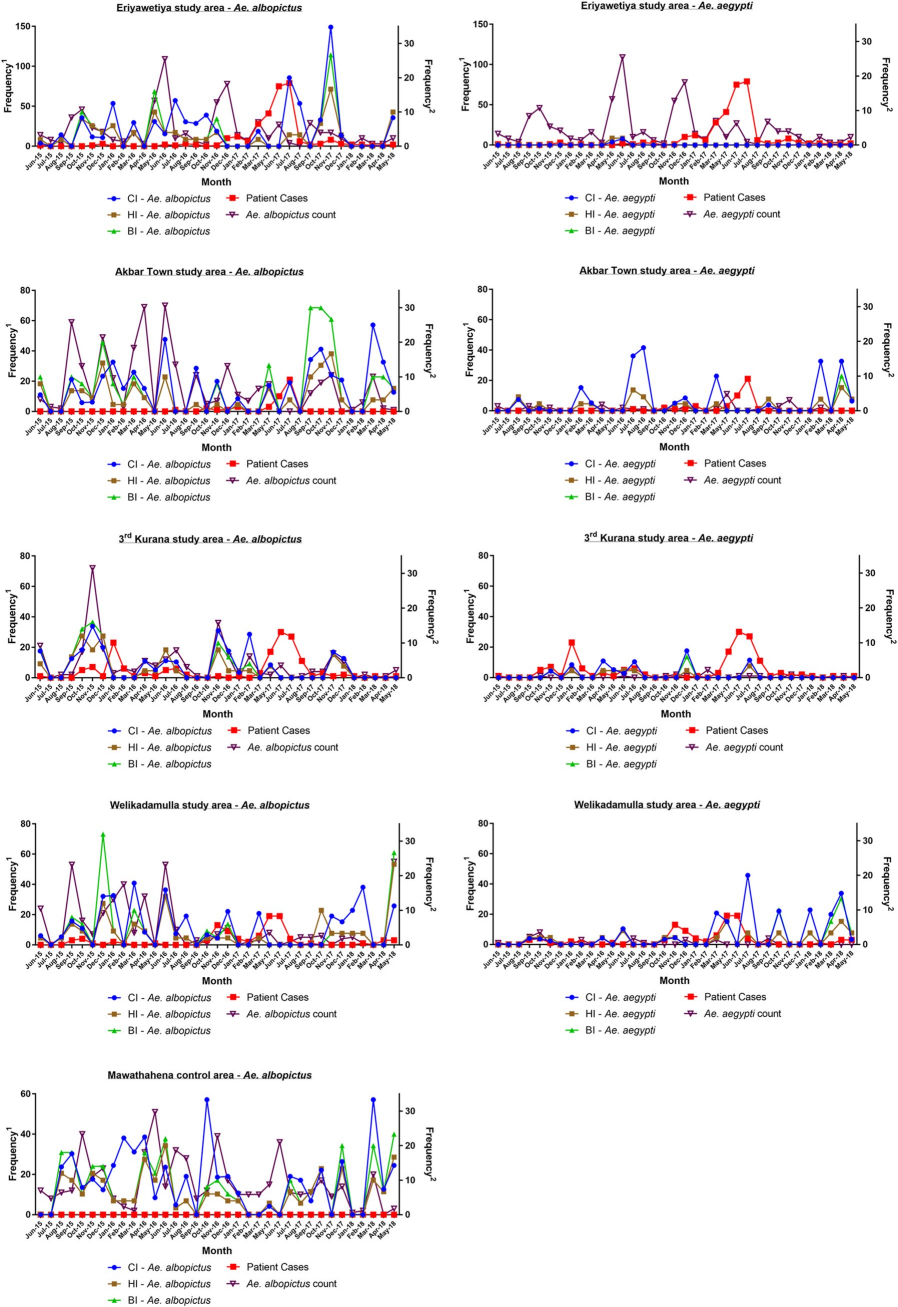
Monthly variation of CI, HI and BI of study areas with dengue vector species and patient cases. Frequency1—Variation of number of incidences and mosquito counts; Frequency2—Variation of CI, HI and BI.

The CI, HI and BI were calculated in monthly basis for *Ae*. *aegpti* and *Ae*. *albopicus* separately for all study areas and plotted against patient count and number of captured mosquitoes (Fig 4).

### Correlations between entomological indices with field-caught dengue vector mosquito counts in the study areas

Cross correlation analysis was performed to identify significant correlations of calculated entomological indices with the count of field-caught adult dengue vector mosquitoes collected from respective species up to three months lag periods. Both CI and HI at zero month lag period were significantly correlated positively with the field-caught *Ae*. *albopictus* mosquito count in the Akbar Town study area. In the 3^rd^ Kurana study area, positive significant correlation was observed with all the indices, CI, HI and BI, with the field-caught *Ae*. *albopictus* mosquito count at zero month lag period. Further, the HI and BI were significantly correlated positively with the field-caught *Ae*. *albopictus* mosquito count at one month lag period in the 3^rd^ Kurana study area. In the welikadamulla study area, positive significant correlation was observed for all the three indices with the field-caught *Ae*. *albopictus* mosquito count at zero month lag period. When considering the *Ae*. *aegypti* field-caught mosquito counts, positive significant correlation was observed only in 3^rd^ Kurana study area with CI and BI at the lag period of two months. No significant correlation between entomological indices and field-caught mosquitoes were observed for Eriyawetiya and Mawathahena (control) areas at 5% significant level (S2 File, Table A4).

### Correlations between entomological indices with reported dengue cases in the study areas

Distribution of dengue incidences depending on gender and age in study and control areas are shown in Table 2. When number of dengue cases in the study areas were compared with control areas, significant correlations of calculated entomological indices with reported patient cases were identified using the cross correlation analysis up to three months lag periods. The significant correlations were summarized in the Table A5 in S2 File. In the analysis, positive significant correlation was observed in HI for *Ae*. *aegypti* for 3^rd^ Kurana and Welikadamulla study areas with reported patient cases at zero and one month lag intervals respectively while none of other indices were not correlated significantly with the reported patient incidences. Since, none of the patient cases were reported from the control area, the Mawathahena study area was excluded for the analysis.

**Table 2:** Distribution of dengue incidences depending on gender and age in study and control areas.

Study area	< 2 Years	3–18 Years	19–39 Years	40–59 Years	60 < Years
**Eriyawetiya**					
Female	3	86	64	29	10
Male	1	85	64	15	6
Total	4	171	125	44	16
**Akbar Town**					
Female	1	9	12	4	3
Male	0	12	8	4	0
Total	1	21	20	8	3
**3**^**rd**^ **Kurana**					
Female	1	45	38	18	7
Male	2	45	30	12	3
Total	3	90	68	30	10
**Welikadamulla**					
Female	3	50	20	8	4
Male	3	41	20	8	1
Total	6	91	40	16	5

### Correlations between entomological findings with climatic variables

Cross correlation analysis was employed to identify significant correlations of calculated entomological indices with studied meteorological variables up to three months lag periods. The correlations were summarized in the Table A6 in S2 File.

When considering correlations of entomological indices with rainfall, positive correlations were observed between current month rainfall and HI and BI of *Ae*. *albopictus* and these correlations were significant in Eriyawetiya, Akbar Town and 3^rd^ Kurana high risk study areas as well as Mawathahena control area. Further, similar significant correlation was observed with HI and BI of *Ae*. *agypti* in Eriyawetiya study area. However, negative correlations were observed with *rainfall*_*t*−3_ in all indices of *Ae*. *albopictus* and these negative correlations were significant in 3^rd^ Kurana study area.

Negative correlations were observed with all indices of *Ae*. *albopictus* with current month number of rainy days in dengue high risk study areas. These negative correlations were significant in Welikadamulla study area with CI of *Ae*. *albopictus*. Further, 3^rd^ Kurana study area also demonstrated a significant negative correlation between HI and BI of *Ae*. *aegypti* and current month number of rainy days. However, control area demonstrated a non-significant positive correlation with current month number of rainy days with all three entomological indices. Non-significant positive correlations were also observed between HI and BI of *Ae*. *aegypti* and current month number of rainy days in Eriyawetiya, Akbar Town and Welikadamulla study areas.

Both minimum and maximum temperatures demonstrated similar correlations with entomological indices. Positive correlations were observed with both three months previous minimum and maximum temperatures. These correlations were significant in Eriyawetiya and 3^rd^ Kurana study areas for HI and BI of *Ae*. *albopictus*. Further, both HI and BI of *Ae*. *albopictus* in 3^rd^ Kurana study area demonstrated a significant positive correlation with previous month and two months previous maximum temperature. However, negative correlations were observed between previous month maximum temperature and all three entomological indices of *Ae*. *aegypti* and these correlations were significant in Welikadamulla study area for HI and BI.

All three entomological indices demonstrated similar correlations with minimum and maximum RH. Negative correlations were observed between previous month and two months previous minimum and maximum RH with all three entomological indices of *Ae*. *albopictus* in all study and control areas. These correlations were significant in 3^rd^ Kurana study area for CI of *Ae*. *albopictus* and Eriyawetiya, Akbar Town and 3^rd^ Kurana study areas for HI and BI of *Ae*. *aegypti*. Similar non-significant negative correlations were observed between one and two months previous minimum RH with HI and BI of *Ae*. *aegypti*.

Negative correlations were observed between all three entomological indices with current month wind speed in all study and control areas. These correlations were significant in Akbar Town study area for all three entomological indices of *Ae*. *albopictus*. Moreover, both HI and BI of both species demonstrated positive correlation with two months and three months previous wind speed and these correlations were significant in 3^rd^ Kurana study area. Futher, similar significant positive correlations were observed between three months previous wnd speed and HI and BI of *Ae*. *aegypti* in Eriyawetiya study area.

### Correlation between field-caught dengue vector mosquitoes with reported patients in the study areas

Cross correlation analysis was used to identify significant correlations between fields caught dengue vector mosquitoes and reported dengue incidences in the study areas with lags up to three months (S2 File, Table A7). During the analysis, significant correlations were observed with number of filed caught adult *Ae*. *aegypti* mosquitoes. Significantly positive correlations were observed at zero and one month lagged time periods with patient cases in Eriyawetiya study area and in Akbar Town study area, similar correlation was observed at two months lag period. No other significant correlations were observed between field collected dengue vector mosquitoes with reported patient cases even though non-significant positive correlations were observed at zero month lagged months in both the 3^rd^ Kurana and Welikadamulla study areas. Since no dengue cases were reported in the control area during the studied period of time, correlations were not studied.

### Correlation between field-caught dengue vector mosquito counts with meteorological variables in the study areas

Cross correlation analysis was used to identify significant correlations between fields caught dengue vector mosquitoes and climatic variables in the study areas up to three months lag periods. Correlations were analyzed with monthly rainfall, number of rainy days, minimum and maximum temperature, minimum and maximum RH, averaged wind speed. The correlations were summarized in the Table A8 in S2 File.

In the analysis, significantly negative correlations were observed with *Rainfall*_*t*−3_, *Rainy days*_*t*−2_, and *Rainy days*_*t*−3_ in total mosquito count and *Ae*. *albopictus* mosquito count in Eriyawetiya study area together with *Maximum RH*_*t*−2_ in *Ae*. *aegypti* mosquito count and *Wind Speed*_*t*−3_ in total mosquito count. Further, significantly positive correlations were observed with *Wind Speed*_*t*−2_ in both the *Ae*. *aegypti* and *Ae*. *albopictus* mosquito count.

In the Akbar Town study area, significantly negative correlations were observed with *Rainfall*_*t*−3_ and *Wind Speed*_*t*−0_ in both total mosquito count and *Ae*. *albopictus* mosquito count and significantly positive correlations were observed with *Maximum RH*_*t*−2_ in *Ae*. *aegypti* mosquito count.

Significantly positive correlations were observed with *Maximum temperatuer*_*t*−2_, *Maximum temperatuer*_*t*−3_, *Maximum RH*_*t*−0_, *Maximum RH*_*t*−1_, and *Wind speed*_*t*−2_ and total mosquito count in 3^rd^ Kurana study area and significantly negative correlations were observed with *Rainfall*_*t*−2_, *Rainy days*_*t*−2_, *Minimum RH*_*t*−2_ and *Maximum RH*_*t*−2_.

Further, significantly positive correlations were observed with *Rainfall*_*t*−0_, *Rainy days*_*t*−0_, *Maximum temperature*_*t*−3_, *Maximum RH*_*t*−0_ and *Wind speed*_*t*−2_ with field-caught *Ae*. *albopictus* mosquito count in the 3^rd^ Kurana study area and significantly negative mosquito correlations were observed with *Rainfall*_*t*−2_, *Rainy days*_*t*−2_, *Minimum RH*_*t*−2_, *Minimum RH*_*t*−3_ and *Maximum RH*_*t*−2_. None of the meteorological variable in 3^rd^ Kurana study area were correlated significantly with the number of filed caught *Ae*. *aegypti* mosquitoes.

During the analysis, only the field-caught *Ae*. *aegypti* mosquitoes were correlated significantly with *Wind speed*_*t*−2_ and none of the other meteorological variable was significantly correlated with the total mosquito count or *Ae*. *aegypti* or *Ae*. *albopictus* mosquito counts in the Welikadamulla study area.

In the Mawathahena control area, both the total mosquito count and the *Ae*. *albopictus* mosquito count were significantly correlated positively with *Rainfall*_*t*−0_, *Rainy days*_*t*−0_, *Minimum RH*_*t*−0_, *Wind speed*_*t*−2_ and *Wind speed*_*t*−3_ and significant negative correlations were observed with the *Rainfall*_*t*−3_, *Rainy days*_*t*−3_, *Maximum RH*_*t*−2_ and *Maximum RH*_*t*−3_. When considering the correlations with *Ae*. *aegypti* mosquito counts, significantly negative correlations were observed with *Minimum temperature*_*t*−2_ and *Wind speed*_*t*−0_.

#### Development of RT-PCR-based assay

**Fig 5:**
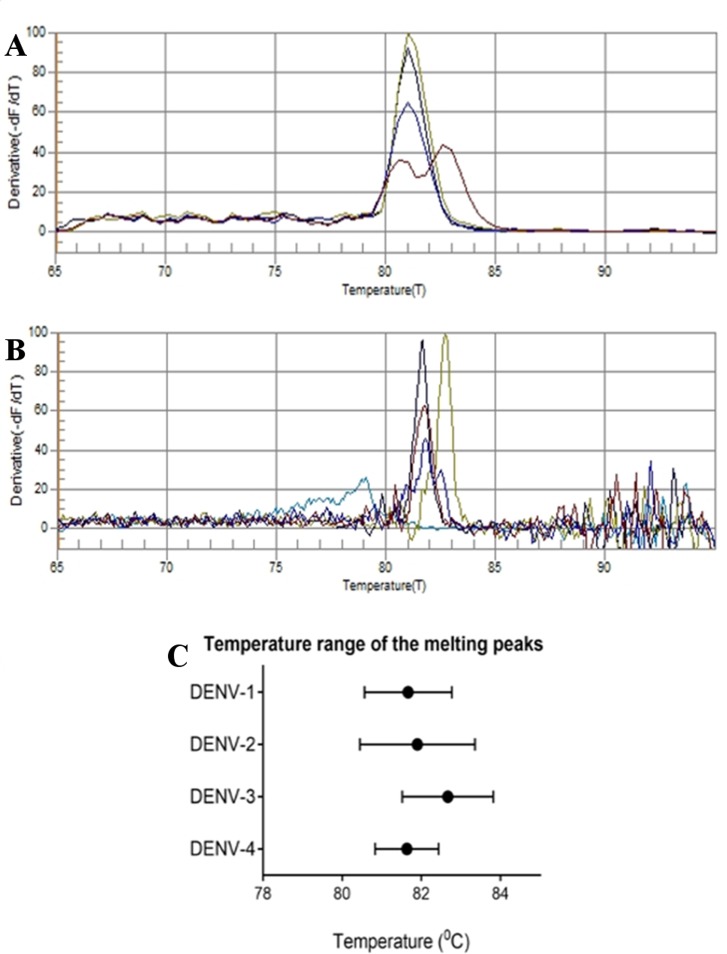
Melting curve analysis of the second PCR. A—Melting curve analysis at 0.4°C step size, B—Melting curve analysis at 0.1°C step size, C—Temperature range of the melting peaks at 0.1°C step size. The colour codes in the first two graphs were Red—DENV-1, Dark Blue—DENV-2, Yellow—DENV-3, Black—DENV-4 respectively.

An rRT-PCR assay was developed to detect presence of DENV in field-caught dengue vector mosquitoes in the study areas using both SYBR Green dye. The resulting fluorescence curve and subsequent melting curve for the first PCR is illustrated in S1 Fig. After the first PCR, semi nested RT-PCR was performed to identify the DENV serotypes using the TS1-4 serotype specific reverse primers. Each reverse primer was added to the master mixture at 0.4 μM concentration. In the RT-PCR assay, the product was 5 times diluted with PCR water prior to addition of 5.0 μl to the reaction mixture as template. The melting peaks that were developed for each DENV serotype following the semi-nested RT-PCR assay was illustrated in the Fig 5. In the melting peak analysis, no clear separation was observed with strong demarcations for the separation of DENV serotypes at the step size of 0.4°C (Fig 5A). However, when the step size reduced to 0.1°C, the range of the developed peaks were differ for the each serotype (Fig 5B) and when analyzing the melting peaks, the ranges of the peaks were 80.6–82.8°C, 80.5–83.4°C, 81.5–83.8°C and 80.8–82.4°C for DENV-1-4 respectively (Fig 5C).

#### Testing of field-caught dengue vector mosquitoes

The field-caught *Aedes* dengue vector mosquitoes were pooled prior to separation of head and thorax of the mosquitoes. Maximum of 50 mosquitoes were pooled together during the preparation of mosquito pools. During the entomological survey, a total of 2,718 adult female dengue vector mosquitoes were collected and out of them, 4.56% (n = 124) of the mosquitoes were *Ae*. *aegypti*. Altogether, 242 mosquito pools were tested using the rRT-PCR assay developed using the primers proposed by Lanciotti *et*. *al*. (1991) and 23.6% (n = 57) of them were *Ae*. *aegypti* pools. The summary of the mosquito pools were mentioned in the Table 3. The highest number of mosquito pools were arranged for *Ae*. *aegypti* from the Akbar Town study area while that of for *Ae*. *albopictus* was from Eriyawetiya high-risk area.

**Table 3:** Summary of adult mosquito pools collected from study and control areas.

Study area	*Ae*. *albopictus* pools (No. of mosquitoes)	*Ae*. *aegypti* pools (No. of mosquitoes)	Total number of pools (No. of mosquitoes)
Eriyawetiya	43 (708)	13 (20)	56 (728)
Akbar Town	39 (601)	17 (46)	56 (647)
3^rd^ Kurana	33 (323)	13 (24)	46 (347)
Welikadamulla	32 (412)	12 (32)	44 (444)
Mawathahena (Control)	38 (550)	2 (2)	40 (552)

**Fig 6:**
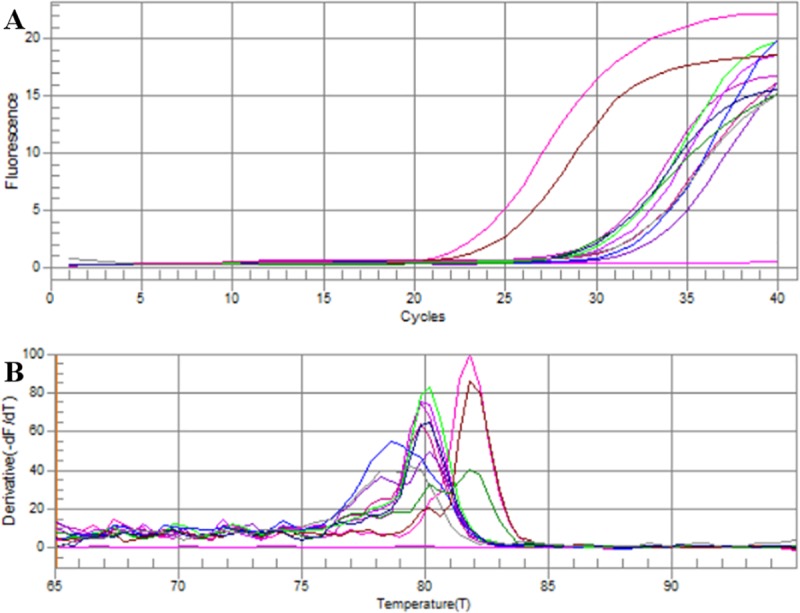
Fluorescence curve and results of subsequent melting curve analysis of the mosquito pools tested on June, 2017. ▂▂ Wattala *Ae*. *aegypti* (n = 1), ▂▂ Wattala *Ae*. *albopictus* (n = 2), ▂▂ Eriyawetiya *Ae*. *aegypti* (n = 4), ▂▂ Eriyawetiya *Ae*. *albopictus* (n = 42), ▂▂ Akbar Town *Ae*. *aegypti* (n = 4), ▂▂ Akbar Town *Ae*. *albopictus* (n = 39), ▂▂ Negombo *Ae*. *albopictus* (n = 2), ▂▂ Negombo *Ae*. *aegypti* (n = 8), ▂▂ Mirigama *Ae*. *albopictus* (n = 37), ▂▂ Mirigama *Ae*. *albopictus* (n = 15), ▂▂ Positive control, ▂▂ Negative control.

During the study, both the *Ae*. *aegypti* and *Ae*. *albopictus* mosquito pools collected on June, 2017 from Eriyawetiya high-risk area were positive DENV and the detected serotype was DENV2. The fluorescence curve of the RT-PCR and the results of melting curve analysis were illustrated in the Fig 6. However, none of the field-caught adult female dengue vector mosquito pools were positive for the presence of DENV.

During the melting curve analysis, the peaks appeared for Eriyawetiya samples were in aligned with the positive control (Fig 6B) at 81.8°C with the range of 80.5 to 81.8°C which is characteristic to the DENV-2 (Fig 6C). Therefore, DENV serotype in the positive samples were identified as DENV-2.

### Genotyping of dengue positive samples

cDNA was prepared using random hexamers from the isolated RNA from positive samples and DENV-2 specific Den2_771F forward primer and Den2_2540R reverse primer were used to completely amplify the E gene. The optimized assay condition used for the PCR was, in 50 μl reaction system, 1x Colorless GoTaq^TM^ reaction buffer, 1.5 mM of MgCl_2_, each dNTP at 0.8 mM, each primer at 0.2 μM with 2.5 U of GoTaq^®^ DNA polymerase with 5–10 μl of cDNA. The optimized thermal profile was initial denaturation at 94.0°C for 10 minutes followed by 40 cycles of denaturation at 94.0°C for 30 seconds, annealing at 58.0°C for 90 seconds and extension at 72.0°C for 2 minutes with final extension at 72.0°C for 7 minutes. The results of agarose gel electrophoresis of the amplified products of genotyping PCR was illustrated in S2 Fig. PCR products were purified using the GeneJET PCR purification kit (Thermo Fisher Scientific, USA) following manufacturer’s instructions, before being sequenced at a commercial facility by using BigDye Terminator v3.1 cycle sequencing kit (Thermo Fisher Scientific, USA).

### Result of sequencing of DENV-2

**Fig 7:**
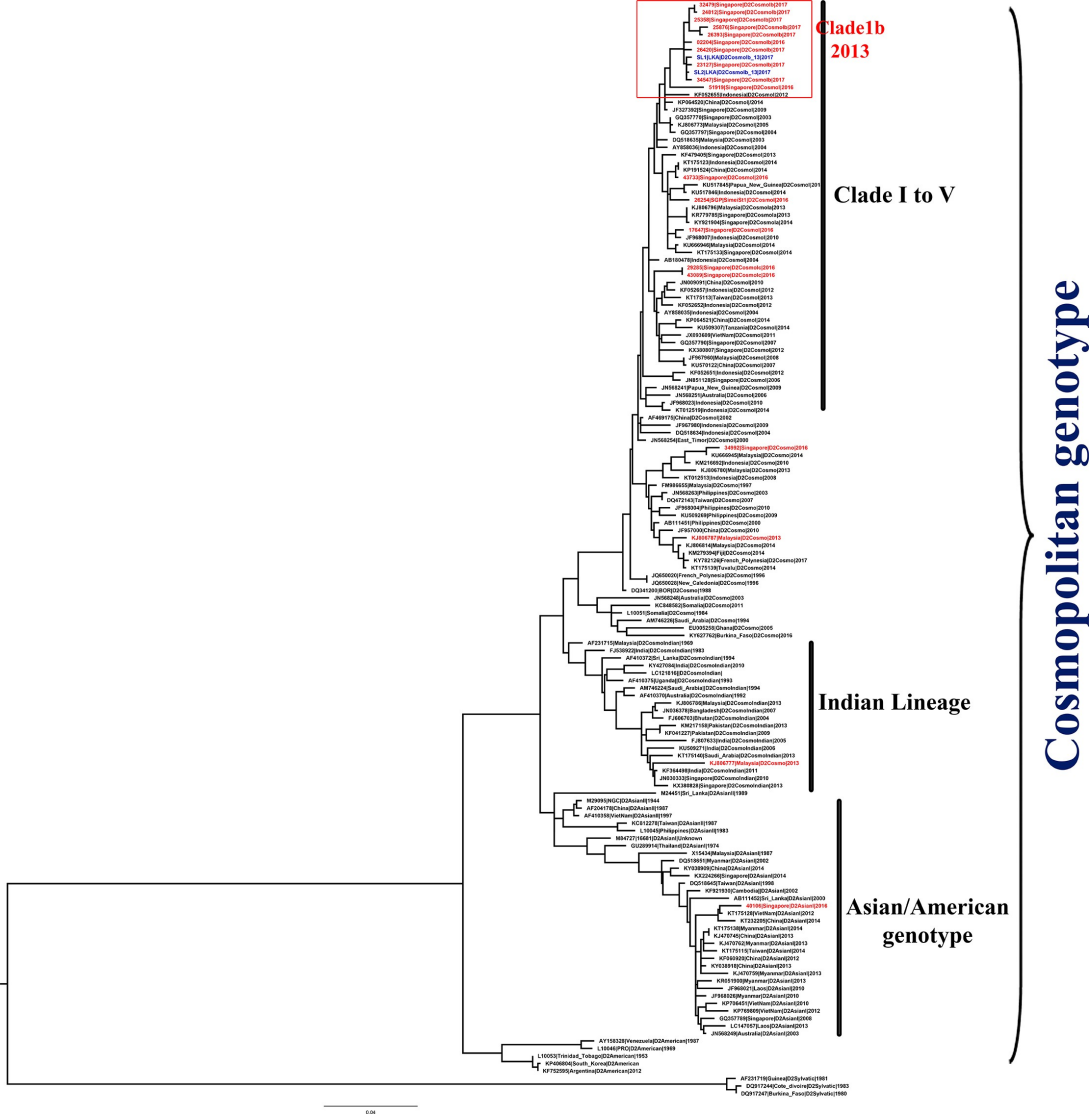
Phylogenetic tree of the DENV-2 based on the E gene sequences. Complete E gene sequences were used to construct the phylogenetic tree with General Time Reversible (GTR) substitution model using the MEGA7 software [26]. Red- DENV-2 sequence from Singapore, Blue- Generated E gene sequences from the positive mosquito pools. Sequences retrieved from GenBank are shown with accession numbers.

After sequencing, the forward and reverse chromatograms generated from the E gene of isolated DENV-2 were assembled in Lasergene 8.00 software suite (DNASTAR Inc., USA). The generated sequences were aligned with retrieved DENV2 sequences available at NCBI database and Consensus sequences were aligned using MAFFT version 7 [22, 23]. The phylogenetic trees were developed using MEGA7 software with General Time Reversible (GTR) substitution model with gamma distributed rates. The robustness of clades was determined by using bootstrap analysis of 500 replicates and the developed phylogenetic tree is illustrated in the Fig 7. During the phylogenetic analysis, the E gene sequence generated from the *Ae*. *aegypti* mosquito pool was denoted as SL1 and the sequence generated from the *Ae*. *albopictus* mosquito pool was denoted as SL2.

The result of the phylogenetic analysis illustrated that the E gene sequences of DENV-2 obtained from the two DENV positive mosquito pools were belong to DENV-2 Cosmopolitan Clade Ib genotype.

### Construction of median joining evolutionary network

Median joining evolutionary network was created to identify mutations of the E gene sequences in the similarly clustered sequences of DENV-2 cosmopolitan Clade Ib genotype to identify and visualize the mutation pattern using the Network 4.6.1 software. The resulted evolutionary network was visualized in S3 Fig. Comparative sequence analysis between the generated DNEV-2 sequences from the mosquito pools and Singapore isolates (2013–17) revealed that the Sri Lankan sequence (SL1) grouped with a sub-clade of clade Ib viruses (variant 3; fixed substitutions—C1383T + T1464C). The genetic relationship of clade Ib viruses between the two countries has been summarized in the following table (Table 4).

**Table 4:** Summary of the genetic relationship of DENV-2 clade Ib viruses between Sri Lankan isolates and Singapore isolates.

Year	Nucleotide composition	Amino Acid
Sequence Identity of Sri Lankan Strain vs.		
2014 (n = 192)	99.2% to 99.7%	99.1% to 100%
2015 (n = 713)	99.1% to 99.7%	99.1% to 100%
2016 (n = 1 216)	99% to 99.7%	99.1% to 100%
2017 (n = 32)	99.3% to 99.7%	99.5% to 100%

According to the results of comparison, sequence analysis, 99.0% to 99.7% genetic similarity was observed between Sri Lankan DENV-2 cosmopolitan Clade 1b isolates to same clade of DENV-2 reported from Singapore during 2016 to 2017 with 99.1% to 100% similarity in amino acid sequences. The evidence suggests recent introduction of this virus strain into Sri Lanka.

### Distribution of DENV-2 Cosmopolitan Clade 1b genotype around the world

Phylogeographic distribution of DENV-2 Cosmopolitan Clade 1b genotype viruses were analysed using the Bayesian Evolutionary Analysis by Sampling Trees (BEAST) software package that utilizes the Bayesian statistical framework to identify the migration pattern of the genotype. Upon completion of the BEAST run, the developed MCC location tree with spatio-tempral information was developed using the TreeAnnotator (version 1.7.4) software tool and visualized using the FigTree software (version 1.4.3) (S4 Fig).

**Fig 8:**
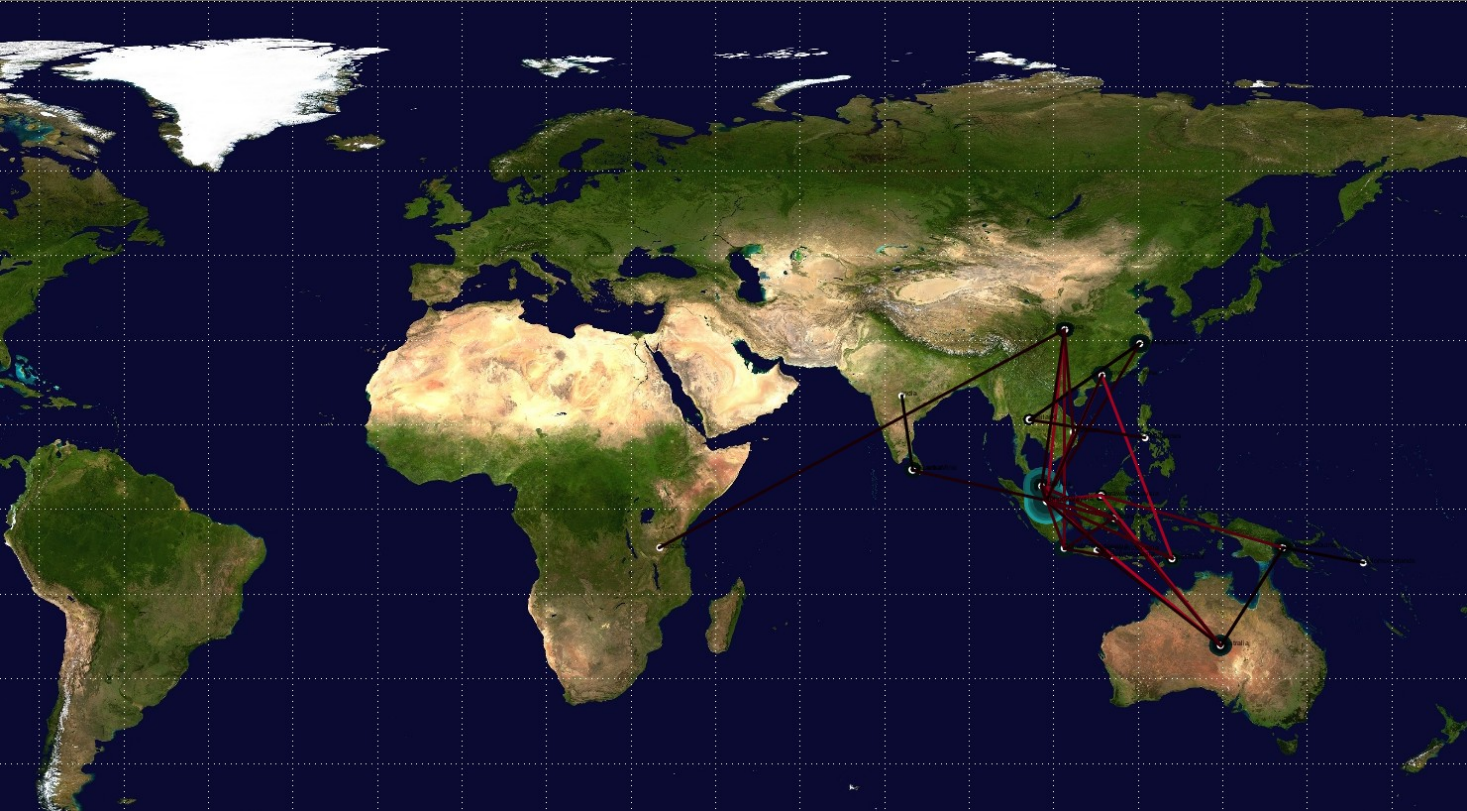
Phylogeographic distribution of the DENV-2 Cosmopolitan Clade 1 genotype in the world. The virus clade had migrated to Sri Lanka from the South-East Asian region. The figure was generated using the map is available in the free software SPREAD [25], which is also available in Maps at CIA (https://www.cia.gov/library/publications/the-world-factbook/images/home_world_map.jpg).

The developed MCC location tree file was then feed to SPREAD (version 1.0.7) software to visualize the phylogeographic output of the MCC tree and the discrete tree phylogenetic module was utilized to generate the.xml file and the location coordinates were fed separately. The results of the phylogeographic analysis confirmed the migration of the DENV-2 Cosmopolitan Clade 1b viruses to Sri Lanka from the South-East Asian region (Fig 8).

### Testing of field-caught immature stages of dengue vector mosquitoes

Mosquito eggs and larvae were pooled separately as mentioned previously in pool preparation of adult mosquitoes during the detection of DENV in the field collected immature stages of *Aedes* dengue vector mosquitoes and the collected larvae of *Aedes* dengue vector mosquitoes were washed with distilled water prior to store them until subjected to RNA extraction. RNA extraction and reverse transcription were performed as mentioned in the Chapter 3.3.1.4.a. The DENV identification PCR was performed using the assay optimized with the primers proposed by Lanciotti *et*. *al*. (1992). The number of the tested dengue vector mosquito larvae and egg pools were mentioned in the Table A9 in S2 File. During the detection of DENV in the field collected immature stages of *Aedes* dengue vector mosquitoes, none of the mosquito pools were positive for DENV.

## Discussion

### Entomological survey

The entomological survey was mainly focused on the collection of adult dengue vector mosquitoes and identification of their breeding places. In total, 10,616 potential breeding places were observed and 2,718 female adult dengue vector mosquitoes were collected during the entomological survey. All the reported mosquito breeding places for immature stages of dengue vector mosquitoes in the control area were positive for *Ae*. *albopictus* and none of them were positive for *Ae*. *aegypti*.

Prior to data analysis, the homogeneity of variances was tested for study variables. During the analysis, variance within the population was significantly different with the number of reported dengue incidences, total mosquito count, field-caught *Ae*. *aegypti* and *Ae*. *albopictus* female adult mosquito counts, dry containers and breeding containers positive for *Ae*. *aegypti* and *Ae*. *albopictus* mosquitoes. However, variances within each population of the number of wet containers and total number of containers positive for immature stages of dengue vector mosquitoes were not significantly different. When considering the number of dengue cases, no incidences were reported during the study period of time in the control area. When considering the control area, only two *Ae*. *aegypti* mosquitoes were captured and none of the breeding places were positive for *Ae*. *aegypti*. The number of field-caught *Ae*. *albopictus* mosquitoes were different in the study and control areas. The highest number of positive breeding places for *Ae*. *albopictus* were identified from the control area and, interestingly, all identified positive containers belonged to *Ae*. *albopictus* in control area (Table 1). Further, the average number of dry containers observed in Akbar Town and Welikadamulla study areas was 1,163 which was 57.3% higher than that of in Eriyawetiya, 3^rd^ Kurana and the control areas (739). Therefore, Tukey's Honestly Significant Difference (Tukey’s HSD) post hoc test [27] was used to identify differences in distribution of wet containers and total number of positive containers between study and control areas. The Games Howell post hoc test [28] was used to identify the differences in total mosquito count, field-caught *Ae*. *aegypti* and *Ae*. *albopictus* female adult mosquito counts, dry containers and breeding containers positive for *Ae*. *aegypti* and *Ae*. *albopictus*. During the analysis, no significant difference was observed between distributions of total field-caught dengue vector mosquito count, *Ae*. *albopictus* count and dry, wet and total positive containers in the studied high risk areas when compared to the control. Significant differences were observed with number of reported dengue incidences, field-caught *Ae*. *aegypti* count, positive containers for *Ae*. *aegypti* and *Ae*. *albopictus* and the differences may be due to the reasons mentioned above [29].

#### Correlations between entomological indices with field-caught dengue vector mosquito counts

The HI is used widely to measure population levels however the index does not consider the effects from the number of positive containers or the productivity of those containers. The CI is also taken in to account only the proportion of water holding containers that are positive for immature stages of dengue vector mosquitoes. Meantime, the BI focus on the relationship between positive containers and households which is the most informative index compared to the HI and CI, however, still the productivity of the containers is not addressed in the BI also.

When considering the all calculated entomological indices of *Ae*. *albopictus*,positive correlation was observed with current month mosquito counts and it was significant in 3^rd^ Kurana and Welikadamulla study areas for all three entomological indices. This observation is probably due to *Ae*. *albopictus* mosquitoes are resting and breeding mainly outdoors and most of their breeding places were identified during the entomological surveillance in the current study. However, non-significant negative correlations were observed with all larval indices of *Ae*. *aegypti* in Akbar Town and Welikadamulla study areas while Eriyawetiya study area demonstrated similar negative correlation was observed with HI and BI. These negative correlations may arise due to uncaptured breeding places present in households, such as some indoor breeding places and water tanks. During the survey, there were some restrictions on searching the inside of households, where mostly *Ae*. *aegypti* mosquitoes are resting, leading to missing some breeding places in those households which is similar to previous observations in Ethiopia China [30, 31].

However, the positive correlations indicated that the mosquito counts increased with the availability of breeding places in the high-risk areas. Therefore, further analysis were performed to identify correlations of entomological indices with reported patient cases and climatic factors.

#### Correlations between entomological findings with climatic variables

When considering correlations between entomological indices and studied meteorological variables, similar correlation pattern was observed in HI and BI. Both the HI and BI demonstrated a positive correlation with current monthly rainfall in all study areas. Even these positive correlations were significant in Eriayawetiya, Akbar Town and 3^rd^ Kurana study areas as well as control area. These positive correlations with current month rainfall were probably due to the availability of breeding containers for *Aedes* dengue vector mosquitoes after rainfalls. However, non-significant negative correlations of CI were observed with current and previous month rainfall Eriyawetiya, Welikadamulla and Mawathahena areas. Further, negative correlations were observed with current month number of rainy days in HI and BI of *Ae*. *albopictus* in dengue high risk areas. These negative correlations were probably due to wash out of the potential containers due to excessive pouring conditions. However, in the control area, non-significant positive correlations were observed with current month rainy days which could be due to higher availability of breeding places for *Ae*. *albopictus* mosquitoes. Further, negative correlations were also observed with two months and three months previous rainfall and number of rainy days in high risk study areas with HI and BI which may be due to reduction of breeding places as a result of evaporation of water in the potential containers, temperature rises and degradation of the quality of water in the water holding containers due to sediments and bacterial activities [32, 33].

When considering temperature, negative correlation was observed between HI and BI of *Ae*. *albopictus* and current month minimum and maximum temperature in most of high risk study areas. These negative correlations of entomological indices is probably due to reduction of ideal breeding places of dengue vector mosquitoes. However, non-significant positive correlations were all three entomological indices of *Ae*. *aegypti* and current month minimum and maximum temperature in high risk study areas. *Ae*. *aegypti* is mainly indoor resting mosquito and *Ae*. *albopictus* is outdoor resting mosquito. In the high risk study areas, temperature ranges from 21.3°C to 34.3°C. In high temperature periods, *Ae*. *aegypti* mosquitoes will fly inside of households and rest in there for their survival and therefore, they can survive better in high temperature periods than *Ae*. *albopictus*. This can be another reason for negative correlations of entomological indices of *Ae*. *albopictus* and positive correlations of *Ae*. *aegypti* entomological indices with temperature. This may indicate that most of *Ae*. *aegypti* mosquitoes are resting indoors in the study areas and therefore, more attention need to be paid to control indoor mosquito populations in future source reduction programmes during epidemic as well as non-epidemic periods. Further, positive correlations were observed between all three entomological indices of *Ae*. *albopictus* and maximum temperature in all study areas at two and three months lag periods. These positive correlations may be due to safeguard of mosquito eggs. During high temperatures, water in the containers gets evaporated and even though the container completely dried out, the eggs can survive without dropping out as the eggs are attached to the walls of the container. Therefore, when there is a rainfall, those eggs can be hatched successfully and develop mosquito larvae leading to positive correlation [34].

When considering the RH, both the minimum and maximum RH were correlated in a similar manner with entomological indices. Positive correlations were observed between current month maximum RH with both HI and BI of *Ae*. *albopictus*. Further, similar non-significant positive correlations were observed between HI of *Ae*. *aegypti* and current month minimum and maximum RH. These positive correlations can be due to the higher RH in study areas. Sri Lanka is an island nation located in the Indian Ocean and a high level of humidity prevails throughout the year. The average RH in Sri Lanka is about 79.8% which makes favorable conditions for the development of dengue vector mosquitoes. Therefore, positive correlation of entomological indices with RH at current month has to be expected in Sri Lanka. However, all three entomological indices of *Ae*. *albopictus* were negatively correlated with two and three month previous minimum and maximum RH in study areas. This may be due to higher RH can reduce the hatching rates of mosquito eggs attached to dry containers which is reported in a previous study [35]. In rearing insectaries, the mosquito eggs were stored in a desiccator below 60% of RH to maximize the hatching rates. This observation indicate that higher RH may be lead to reduce hatching rates of mosquito eggs. When the eggs were attached outdoors at high level of RH for long periods, the hatching rates will be decreased leading to a negative correlation of the entomological indices with two and three month previous minimum and maximum RH.

When considering the wind speed, negative correlations were observed between all three entomological indices of *Ae*. *albopictus* and current month average wind speed and these correlations were significant in Akbar Town study area. The negative correlations may be explained by the difficulties in finding breeding places as well as hosts for dengue vector mosquitoes due to suppressing of mosquito flights at higher wind speed. However, non-significant positive correlation was observed in HI and BI of *Ae*. *aegypti* with current month wind speed in Akbar Town and 3^rd^ Kurana study area. The reason may be attributed to the indoor resting of *Ae*. *aegypti* mosquitoes. Since the *Ae*. *aegypti* mosquitoes are resting indoors, they can find a host easily for their blood feedings. Further, the *Ae*. *aegypti* mosquitoes are reluctant to fly long distances and they prefer artificial breeding containers close to their resting household. In the entomological survey, many potential artificial breeding places were identified and *Ae*. *aegypti* mosquitoes can find better breeding container with the aid of wind speed. Meantime, positive correlations were observed between HI and BI of both the species with two months previous average wind speed and it was significant in 3^rd^ Kurana study area for both the species and Eriyawetiya study area for *Ae*. *aegypti*. These positive correlations may be explained by migration of dengue vector mosquitoes by air waves and wind. Even though the movements of *Ae*. *aegypti* was greatly affected by human activities, previous studies have mentioned that the wind currents may also advances the movements of large populations of mosquitoes [36]. These positive correlations at lag 2 and 3 months of wind speed suggested that the possibility of movement of dengue vector mosquitoes from another area to study areas and emphasized areas of attention that has to be paid intensively during future vector control programmes [37].

#### Correlations between entomological indices with reported dengue cases in the study areas

No significant correlations were observed with entomological indices of *Ae*. *albopictus* and reported patient cases. However, non-significant negative correlations were observed in the Eriyawetiya study area. This may be due to other vector control and source reduction activities conducted by the MOH office and residents in the study areas when there were dengue incidences. The Eriyawetiya study areas was highly affected by dengue and it was the highest number of dengue incidences reported study area among all study areas. The breeding places of dengue vector mosquitoes got reduced at the time of field surveys as the dwellers in the area removed potential breeding places from their households which lead to the reduction of potential breeding places of dengue vector mosquitoes even though the reported cases were higher [38, 39]. Even though adult mosquitoes are the vectors of DENV, those entomological indices focus on immature stages of dengue vector mosquitoes. During vector control activities, some of the breeding places may be unidentified depending on level of personal expertise, restrictions of accessibility to household, limitations due to inadequate resources, etc. which lead to differences between actual adult population and larval indices. Although those possibilities were addressed in the current study, some restrictions were aroused during investigation in inside of households. This may lead to poor correlation between entomological indices and dengue incidences in the study areas.

### Molecular identification of DENV transmitted by dengue mosquito vectors

Field-caught mosquitoes were tested by rRT-PCR assay. Initially, conventional RT-PCR and semi-nested PCR assays were established. Then rRT-PCR was developed to detect and type DENV in field-caught mosquito samples.

#### Development of rRT-PCR assay

The real-time assays were developed using both SYBR Green and EvaGreen fluorescence dyes. The assays were developed initially with the QuantiTech SYBR Green PCR kit. During the melting analysis in the first rRT-PCR developed with the primers proposed by Lanciotti *et*. *al*. (1992), the melting peaks for DENV-2-4 appeared around 82°C while a broad melting peak appeared for DENV-1. The broad peak appeared in DENV-1 may be due to the variations in the GC content at the 5’ and 3’ ends or binding differences of SYBR Green of the amplified products of DENV-1. During the detection of serotypes, demarcations of serotypes were not strong at low level resolution (steps of 0.4°C). However, when using high melting resolutions with 0.1°C step size, different peak ranges were observed as illustrated in the Fig 4.15. However, SYBR Green is a non-saturation fluorescence dye and it binds to dsDNA randomly. Since the size of the amplified product was 511 bp in length there is higher probability that the florescence curve developed from SYBR Green was not correlated proportionally with the amplified product as it can get attached to anywhere in the dsDNA.

#### Testing of field-caught dengue vector mosquitoes to detect DENV

The field-caught mosquitoes were tested using the developed rRT-PCR assay with primers developed by Lanciotti *et al*. (1992). Wings and legs were removed and both head and thorax subjected for the RNA extractions. During the RNA extraction procedure, special precautions were taken to minimize the degradations by RNase enzyme. Precautions were taken into account during the preparation of buffers and solutions as well as throughout the extraction procedure. All glassware and crushing rods were rinsed thoroughly with DEPC treated water and autoclaved to remove residual DEPC. Molecular grade reagents were used to prepare stock solutions and the buffers were freshly prepared. Further, separate bench spaces, pipette sets and personal protection measures were followed.

During testing of mosquito pools, pools prepared with collected mosquitoes from Eriyawetiya study areas in June, 2017 were positive for the DENV. In June and July, 2017, Sri Lanka experienced the largest dengue epidemic reported so far with 110,370 dengue incidences with 301 dengue related deaths in the first seven months of the year 2017 [40]. The epidemic peak was observed in the July, 2017 and during the epidemic peak, the highest number of dengue incidences were reported from the District of Gampaha [6]. When considering the reported dengue incidences in the Eriyawetiya study area, 41, 75 and 79 dengue incidences were reported in May, June and July months in 2017 respectively. During the entomological survey in June, 2017, 27 *Ae*. *albopictus* and 4 *Ae*. *aegypti* mosquitoes were collected and both the *Ae*. *aegypti* and *Ae*. *albopictus* mosquito pools were positive for DENV. When detecting the infected serotypes, both the samples belonged to DENV2. These mosquitoes may infect DENV through horizontal transmission as there was a severe outbreak at study areas during this period of time.

None of the other *Aedes* mosquito pools were positive for DENV in Eriyawetiya study area or other study and control areas. Further, none of the *Aedes* dengue vector mosquito eggs and larvae pools tested to detect vertical transmission of DENV were positive. The level of vertical transmission of DENV is rarely detected in the field even though the phenomenon is an important strategy to explain arboviral disease transmission in nature [41, 42].

#### Importance of phylogeographic analysis

The genotyping PCR was performed for DENV positive samples to amplify complete E gene using the DENV2 specific primers. The derived E gene sequences generated from the positive mosquito pools were compared with the available DENV-2 gene sequences at NCBI GenBank database and multiple sequence alignment was prepared using the MAFFT 7 software which utilize the Fast Fourier transformation which generate the alignment faster and accurately. Substitution models are used to predict the rate of substitution for nucleotides at a given site together with the distribution of substitutions across the entire sequence during generation of phylogenetic trees. The GTR model, which is also known as the General Reversible (REV) model, is more complex type of models that assumes different rates of substitution for each pair of nucleotides, in addition to assuming different frequencies of occurrence of nucleotides. The result of the phylogenetic analysis illustrates that the E gene sequences of DENV2 obtained from two DENV positive mosquito pools belong to DENV2 Cosmopolitan Clade Ib. The virus strain was detected in Singapore since 2013 and it has been the dominant strain in South-East Asia, specially Singapore, Indonesia, Malaysia and China since August, 2015. Comparative sequence performed using network indicate that the sequences generated from the mosquito pools were grouped with a Singapore isolates (2013–17) sub-clade variant 3 of clade Ib viruses which exhibits fixed substitutions of C1383T and T1464C, which caused the latest DENV-2 outbreak in Singapore during early 2016. Further, the BEAST analysis also indicated that the DENV clade Ib virus has migrated to Sri Lanka from the South-East Asian region. These evidences suggested recent introduction of this DENV strain to Sri Lanka and the cause for the epidemic in mid-2017. The DENV-2 clade Ib virus was reported to be the dominant viral strain since August, 2015 and it migrated to Sri Lanka approximately 2 years’ later. Since the virus strain was introduced to Sri Lanka recently and there was no herd immunity against the viral strain, the strain spread throughout the country increasing the number of dengue incidences rapidly until it reached an epidemic level. If there was a system in Sri Lanka to detect such migrations of virulent strains of DENV, the magnitude of the severity of the outbreak could be minimized with the aids of previous reports and literatures because most of times when there is an epidemic in an areas, the viral serotypes and genotypes as well as its virulence were reported to many WHO databases and online resources. Therefore, once a virulent genotype found, using the phylogeographic model, it is possible to analyze its spread and virulence at early stage of outbreak locally and control it before propagating into an epidemic. Furthermore, once the data gathered over few years, it is possible to identify recurrence of previously identified viral genotypes over the periods. This is mainly observed in after an epidemic. During an epidemic, most of times a viral genotype predominates other serotypes and genotypes when these epidemic strains introduced to the community recently. Therefore, there will be no enough herd immunity against the viral strain leading to rapid spread of the strain. Once the epidemic is over, where the herd immunity for the disease is mainly replaced by the epidemic strain, there is a possibility to overtake other already existing virulent DENV strains after a few years of silence. Since the population does not have enough herd immunity against those strains now, there is a higher possibility of another dengue outbreak, probably at lower magnitude as there is some level of herd immunity against the strain already in the population, due to reoccurrence of previously existing epidemic strains. These scenarios can be tracked using the developed phylogeographic model when it applied locally over some years and minimize spread of outbreaks at early stage.

District of Gampaha is located next to the District of Colombo, the capital of Sri Lanka, where the highest number of dengue cases are reported every year. Previous studies mentioned that during the daily commute of residents in the District of Gampaha to the District of Colombo for work and other commercial purposes, they can be infected by dengue. However, these patients are reported under the District of Gampaha [9]. There is no proper mechanism to track these commute related infections so far in the country. Further, it is a great concern that the dengue cases were reported sporadically prior to its spread all over the country. The proposed phylogenetic approach can be the solution to detect migration patterns of DENV serotypes throughout the country. For example, when cases are reported from a remote area, the generated E gene sequences can be modelled to identify the similarities and mutation patterns and compared with the sequences from outbreak areas to detect a possible migration pattern. Therefore, it is possible to identify whether the same DENV strain has spread to new areas. This may lead to identification of significant mutations for successful proliferations of DENV and it is reported that most of the generated mutations in the DENV strains are not epidemic while epidemic strains successfully proliferate in the environment causing outbreaks and epidemics [43]. In the scenario, the Colombo and Gampaha districts should not be considered separately as it can hinder many important evidences.

The fifth serotype of DENV has already been reported [44, 45]. If the serotype migrated to Sri Lanka where no prior cases are reported from the serotype and so no herd immunity, there is higher possibility of dengue epidemic depending on the virulence of the strain. A phylogeographyic model, like the one developed in the study, can detect the origin and distribution of the strains which will minimize the establishment of new DENV strains locally. This will enable utilize scare public health resources efficiently to minimize magnitudes of impending dengue epidemics.

## Conclusions

In the study, we identified a migrated DENV serotype 2, which belongs to the cosmopolitan Clade1b genotype leads the dengue epidemic in July, 2017 in Sri Lanka. This viral strain is the predominant strain in South-East Asian region since 2015. A real-time model of this approach can identify risks of virulent forms of DENV in early stage in individual clusters. Since the epidemiology and virulence of the DENV strain is known from previous areas, the spread of the disease can be minimized in the new area with more specified vector control and source reduction programmes managing scarce public health resources effectively. When the system is established in all areas, then it will be easy to identify the pattern of migrations of newly introduced DENV strains over the time in the country.

## Supporting information

S1 Fig Melting curve analysis of the first PCR. A - DENV-1, B - DENV-2, C - DENV-3, D - DENV-1. In the analysis, melting peak was detected for DENV-1 at 83.4°C and, for the rest of the serotypes, the peak detected around 82°C.
(TIF)

S2 Fig Agarose gel electrophoresis amplified product of the complete E gene. 1 - *Ae*. *aegypti* pool 1, 2 - *Ae*. *albopictus* pool 2, 3 - Negative control, 4 - 100 bp DNA ladder.
(TIF)

S3 Fig Median joining network analysis of E gene sequences of DENV-2 Cosmopolitan Clade 1b genotype sequences. (TIF)

S4 Fig MCC location tree developed with spatio-temporal information by the TreeAnnotator. The colours represents the countries and the clades highlighted in yellow colour indicates SL1 and SL2 DENV-2 Cosmopolitan Clade 1b sequences.
(TIF)

S1 File Formulae employed for the calculation of Entomological Indices. House index (HI), Container index (CI) and Breteau index (BI) were calculated following these formulas.
(DOCX)

S2 File Supporting tables. (DOCX)

S3 File Accession numbers of the E-gene sequences used for phylogenetic analysis. (XLSX)
