# Supplementary figures and images for: Entomological surveillance with viral tracking demonstrates a migrated viral strain caused dengue epidemic in July, 2017 in Sri Lanka
Source: PLoS One. 2020 May 6;15(5):e0231408. doi: 10.1371/journal.pone.0231408 (PMC7202666; doi:10.1371/journal.pone.0231408)

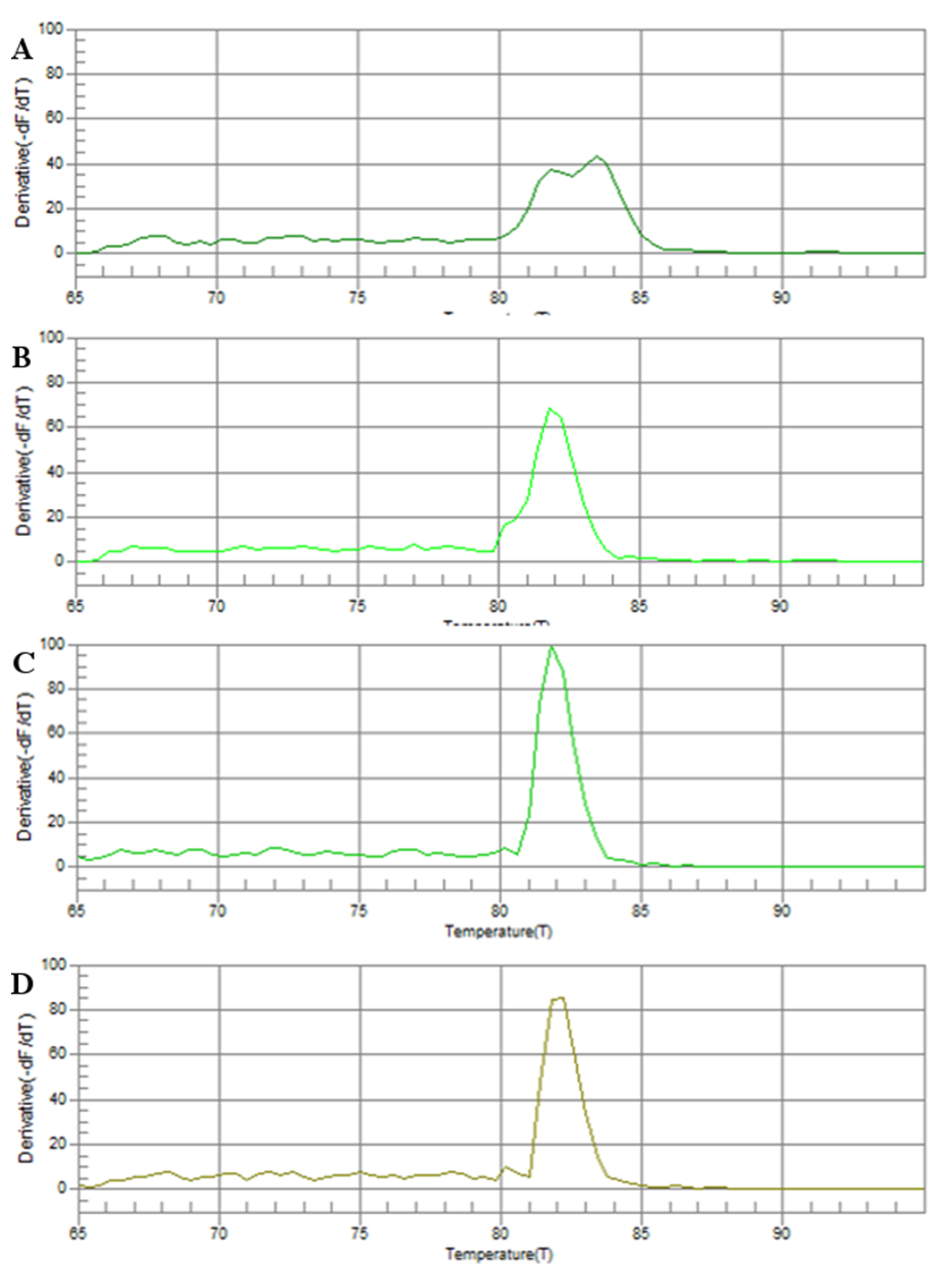

Supplement: S1 Fig — A - DENV-1, B - DENV-2, C - DENV-3, D - DENV-1. In the analysis, melting peak was detected for DENV-1 at 83.4°C and, for the rest of the serotypes, the peak detected around 82°C. (TIF) [file pone.0231408.s001.tif]

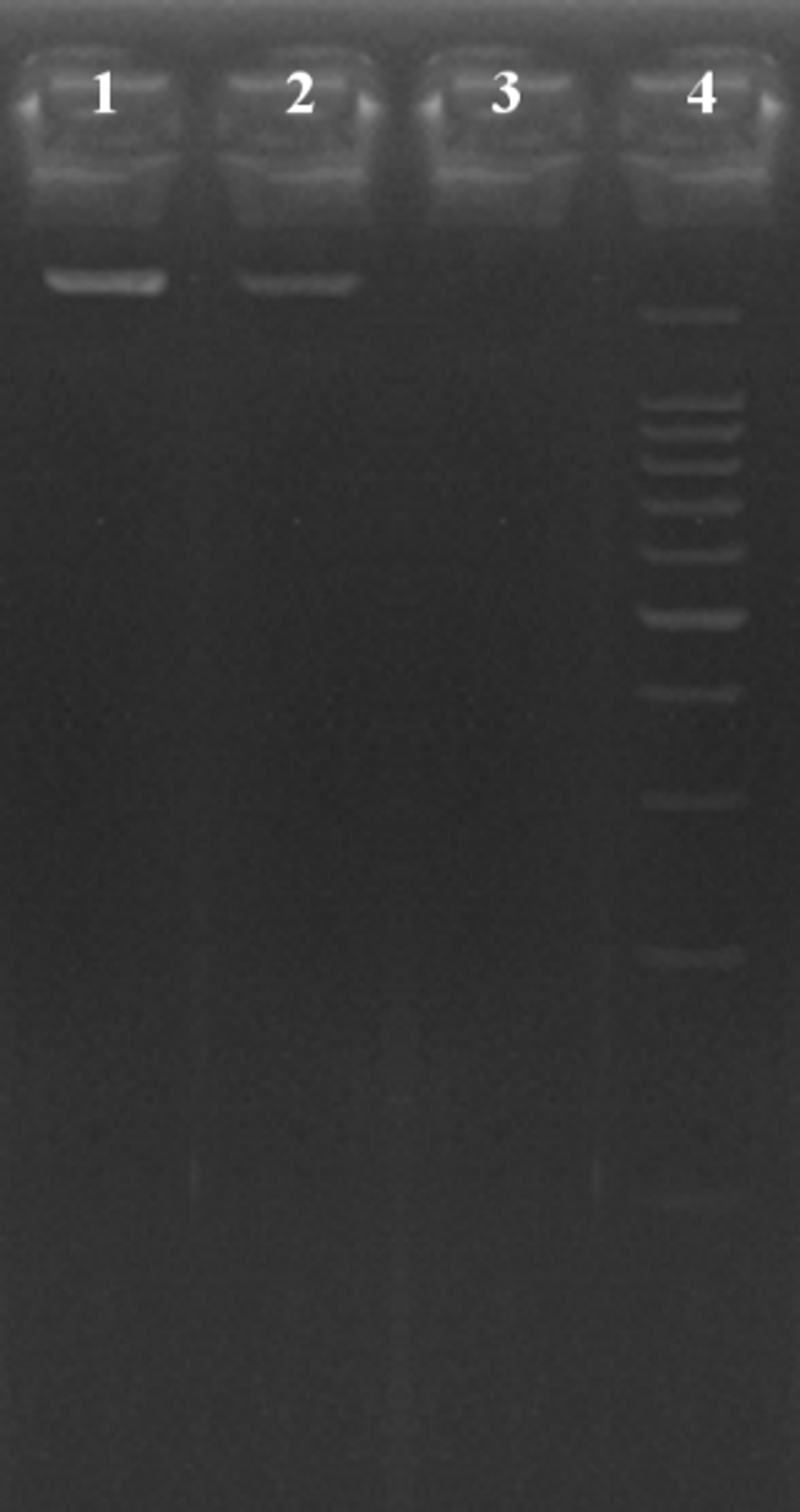

Supplement: S2 Fig — 1 - Ae. aegypti pool 1, 2 - Ae. albopictus pool 2, 3 - Negative control, 4 - 100 bp DNA ladder. (TIF) [file pone.0231408.s002.tif]

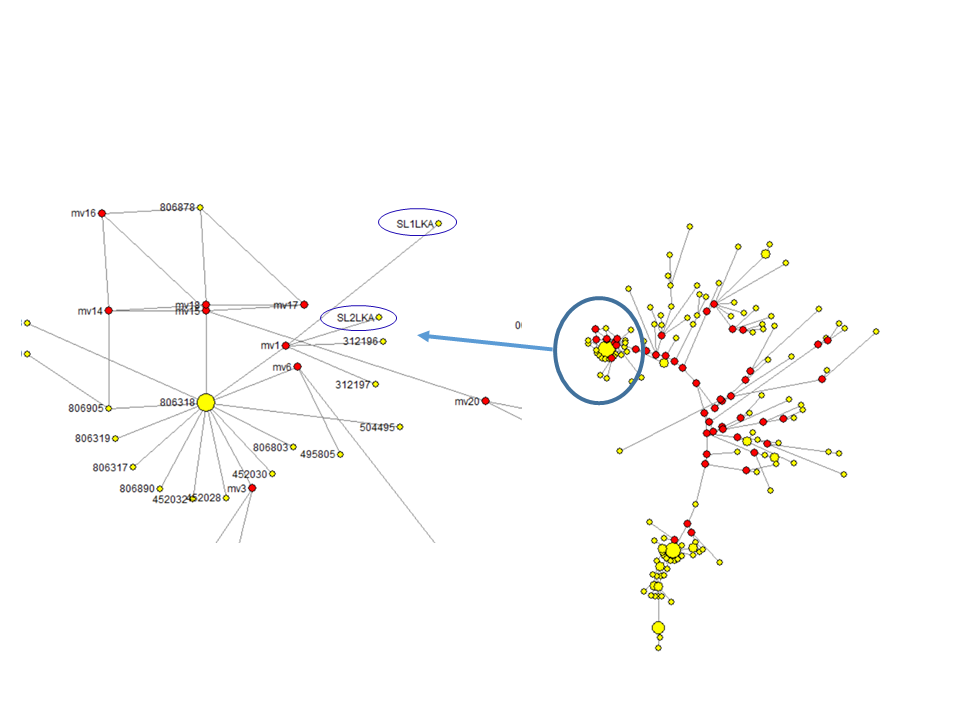

Supplement: S3 Fig — (TIF) [file pone.0231408.s003.tif]

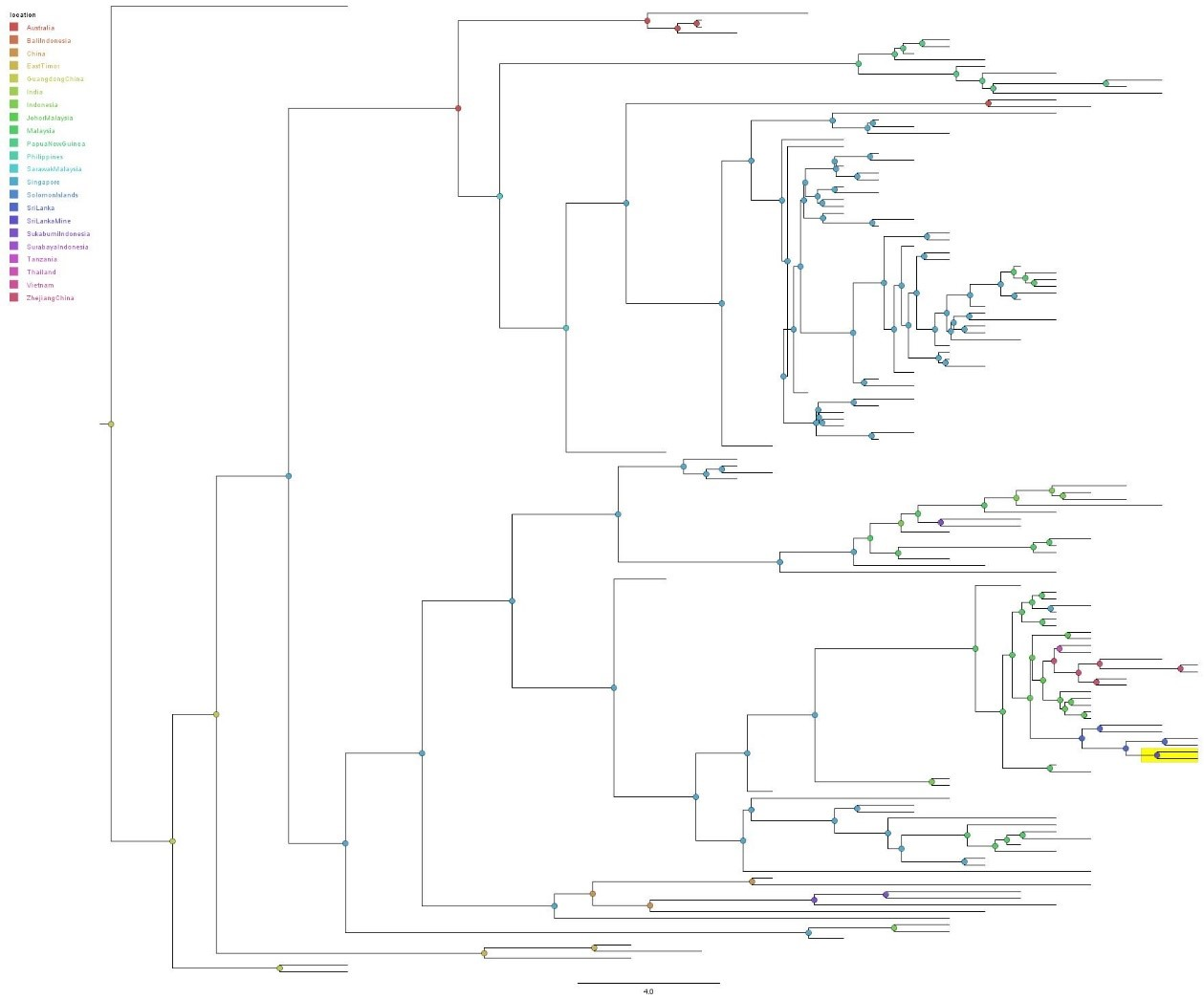

Supplement: S4 Fig — The colours represents the countries and the clades highlighted in yellow colour indicates SL1 and SL2 DENV-2 Cosmopolitan Clade 1b sequences. (TIF) [file pone.0231408.s004.tif]
