# Supplementary material for: Entomological surveillance with viral tracking demonstrates a migrated viral strain caused dengue epidemic in July, 2017 in Sri Lanka
Source: PLoS One. 2020 May 6;15(5):e0231408. doi: 10.1371/journal.pone.0231408 (PMC7202666; doi:10.1371/journal.pone.0231408)
Supplement: S1 File — House index (HI), Container index (CI) and Breteau index (BI) were calculated following these formulas. (DOCX) [file pone.0231408.s005.docx]

House index (HI): percentage of houses infested with larvae and/or pupae.

$$HI= \frac{Infested houses x 100}{Total number of inspected houses}$$

Container index (CI): percentage of water-holding containers infested with larvae or pupae.

$$CI= \frac{Positive containers x 100}{Total number of inspected containers}$$

Breteau index (BI): number of positive containers per 100 houses inspected.

$$BI= \frac{Number of postive containers x 100}{Total number of inspected houses}$$
