# Supplementary material for: Entomological surveillance with viral tracking demonstrates a migrated viral strain caused dengue epidemic in July, 2017 in Sri Lanka
Source: PLoS One. 2020 May 6;15(5):e0231408. doi: 10.1371/journal.pone.0231408 (PMC7202666; doi:10.1371/journal.pone.0231408)
Supplement: S2 File — (DOCX) [file pone.0231408.s006.docx]

Table A1. Primer sequences (Lanciotti *et al*.,1992) used for PCR and semi-nested PCR

| **Primer** | **Sequence** | **Target band sizes (bp)** |
| --- | --- | --- |
| **Forward primer** | | |
| D1 | 5'-TCAATATGCTGAAACGCGCGAGAAACCG-3' |  |
| **Reverse primers** | | |
| D2 | 5'-TTGCACCAACAGTCAATGTCTTCAGGTTC-3' | 511 |
| TS1 | 5'-CGTCTCAGTGATCCGGGGG-3' | 482 |
| TS2 | 5'-CGCCACAAGGGCCATGAACAG-3' | 119 |
| TS3 | 5'-TAACATCATCATGAGACAGAGC-3' | 290 |
| TS4 | 5'-CTCTGTTGTCTTAAACAAGAGA-3' | 392 |

Table A2. Primer sequences used for genotyping

| **Primer** | **Sequence** | **Target band sizes (bp)** |
| --- | --- | --- |
| **Serotype 1** | | |
| Den1_819F | 5'- TGAGACACCCAGGATTCAC -3' | 1779 |
| Den1_2598RN | 5'- GCTGATCGWATTCCACACAC -3' |  |
| **Serotype 2** | | |
| Den2_771F | 5'- ACATGGATGTCRTCAGAAGG -3' | 1769 |
| Den2_2540R | 5'- GAAGGGGATTCTGGTTGG -3' |  |
| **Serotype 3** | | |
| Den3_793F | 5'- AGTCGAGAAGTAGAGACATGG -3' | 1710 |
| Den3_2503R | 5'- CTCTGTCCAGGTGTGGACCT -3' |  |
| **Serotype 4** | | |
| Den4_613F | 5'- ATYCCGAACCTGAAGACATTGA -3' | 1906 |
| Den4_2519R | 5'- TACTGRTCTGTCCAAGTGTGCAC -3' |  |

Table A3. Summary of breeding places positive for immature stages of dengue vector mosquitoes

| Type of breeding place | No. of containers positive for dengue vector mosquito larvae in study areas | | | | | | | |
| --- | --- | --- | --- | --- | --- | --- | --- | --- |
|  | Eriyawetiya | Akbar Town | 3rd Kurana | | Welikada-mulla | | Mawatha-hena | |
| Natural breeding places | | | | | | | | |
| Alocasia plant | 0 | 1 | | 0 | | 3 | | 3 |
| Banana leaves | 2 | 1 | | 1 | | 4 | | 0 |
| Bamboo stump | 0 | 6 | | 0 | | 0 | | 3 |
| Bromilia plant | 0 | 0 | | 1 | | 0 | | 1 |
| Bush stump | 5 | 9 | | 0 | | 2 | | 13 |
| Coconut shell | 3 | 0 | | 4 | | 0 | | 1 |
| Dried leaves | 0 | 0 | | 0 | | 0 | | 1 |
| Dried banana leaves | 0 | 4 | | 0 | | 1 | | 1 |
| Pine-apple plant | 1 | 0 | | 0 | | 1 | | 4 |
| Plant axils | 6 | 4 | | 5 | | 1 | | 23 |
| Tree hole | 0 | 2 | | 1 | | 1 | | 6 |
| Man-made breeding places | | | | | | | | |
| Aluminum pot | 2 | 2 | | 1 | | 1 | | 5 |
| Bird bath | 0 | 1 | | 1 | | 0 | | 0 |
| Cement pot | 0 | 0 | | 1 | | 0 | | 0 |
| Cement tank | 0 | 0 | | 1 | | 0 | | 1 |
| Ceramic cistern | 1 | 0 | | 0 | | 0 | | 0 |
| Ceramic cup | 0 | 1 | | 0 | | 0 | | 0 |
| Ceramic pot | 0 | 0 | | 0 | | 0 | | 1 |
| Ceramic ware | 0 | 1 | | 2 | | 0 | | 0 |
| Concrete slab | 0 | 1 | | 0 | | 0 | | 0 |
| Earthen pot | 6 | 0 | | 5 | | 3 | | 6 |
| Earth pipe | 0 | 0 | | 0 | | 0 | | 1 |
| Fish tank | 0 | 3 | | 0 | | 2 | | 0 |
| Flower pot | 1 | 0 | | 2 | | 5 | | 3 |
| Flower pot tins | 1 | 0 | | 0 | | 0 | | 0 |
| Glass bottles | 1 | 5 | | 0 | | 0 | | 1 |
| Machinery parts | 0 | 0 | | 0 | | 1 | | 0 |
| Metal barrel | 0 | 0 | | 0 | | 1 | | 1 |
| Metal cup | 0 | 0 | | 1 | | 2 | | 0 |
| Metal parts | 1 | 0 | | 0 | | 0 | | 0 |
| Metal pipe | 0 | 0 | | 1 | | 0 | | 0 |
| Metal plate | 0 | 2 | | 0 | | 0 | | 0 |
| Pet dish | 2 | 7 | | 2 | | 3 | | 3 |
| Plastic and polythene items | 18 | 41 | | 11 | | 17 | | 30 |
| Pond | 0 | 0 | | 0 | | 0 | | 1 |
| Refrigerator cover | 0 | 0 | | 0 | | 1 | | 0 |
| Rexine cover | 0 | 1 | | 1 | | 0 | | 0 |
| Roof gutter | 0 | 1 | | 0 | | 1 | | 1 |
| Tin | 1 | 4 | | 5 | | 2 | | 1 |
| Tube well | 0 | 0 | | 4 | | 7 | | 0 |
| Tyre | 4 | 11 | | 3 | | 23 | | 5 |
| Yogurt cup | 0 | 0 | | 2 | | 0 | | 0 |

Table A4. Summary of correlations of entomological indices with field-caught dengue vector mosquitoes in the study areas.

| Entomological index | *Ae. albopictus* count | | | | *Ae. aegypti* count | | | |
| --- | --- | --- | --- | --- | --- | --- | --- | --- |
|  | Lag0 | Lag1 | Lag2 | Lag3 | Lag0 | Lag1 | Lag2 | Lag3 |
| Eriyawetiya | | | | | | | | |
| CI | -0.085 | -0.030 | -0.078 | -0.036 | 0.026 | -0.068 | 0.017 | -0.077 |
| HI | 0.171 | 0.182 | -0.168 | -0.159 | -0.020 | -0.025 | -0.029 | -0.034 |
| BI | 0.205 | 0.277 | -0.183 | -0.162 | -0.020 | -0.025 | -0.029 | -0.034 |
| Akbar Town | | | | | | | | |
| CI | 0.419* | -0.067 | -0.240 | 0.279 | -0.102 | 0.098 | -0.105 | -0.186 |
| HI | 0.456* | 0.015 | -0.290 | 0.213 | -0.202 | 0.126 | -0.022 | -0.166 |
| BI | 0.299 | -0.034 | -0.261 | 0.076 | -0.196 | 0.082 | -0.018 | -0.140 |
| 3^rd^ Kurana | | | | | | | | |
| CI | 0.820* | 0.252 | -0.046 | -0.173 | 0.139 | -0.172 | 0.386* | -0.221 |
| HI | 0.611* | 0.522* | 0.101 | -0.193 | 0.137 | -0.161 | 0.250 | -0.262 |
| BI | 0.798* | 0.478* | 0.045 | -0.195 | 0.123 | -0.166 | 0.496* | -0.243 |
| Welikadamulla | | | | | | | | |
| CI | 0.380* | 0.122 | -0.217 | 0.358* | -0.122 | -0.151 | 0.049 | -0.186 |
| HI | 0.669* | 0.059 | -0.027 | 0.062 | -0.098 | -0.022 | -0.103 | -0.165 |
| BI | 0.571* | 0.170 | 0.123 | 0.079 | -0.137 | -0.091 | -0.112 | -0.136 |
| Mawathahena (Control area) | | | | | | | | |
| CI | 0.005 | 0.229 | 0.069 | 0.203 | - | - | - | - |
| HI | 0.179 | 0.273 | 0.041 | -0.034 | - | - | - | - |
| BI | 0.163 | 0.207 | -0.009 | -0.025 | - | - | - | - |

* Significantly correlated lagged variables with mosquito count of respective species during the study period. A 5% significance level was used to identify significant correlations.

Table A5. Summary of correlations of entomological indices with reported patient cases in the study areas.

| Entomological Index | *Ae. albopictus* count | | | | *Ae. aegypti* count | | | |
| --- | --- | --- | --- | --- | --- | --- | --- | --- |
|  | Lag0 | Lag1 | Lag2 | Lag3 | Lag0 | Lag1 | Lag2 | Lag3 |
| Eriyawetiya | | | | | | | | |
| CI | 0.142 | -0.181 | -0.174 | -0.156 | -0.088 | -0.091 | -0.082 | -0.085 |
| HI | -0.133 | -0.205 | -0.206 | -0.211 | -0.098 | -0.094 | -0.091 | -0.087 |
| BI | -0.121 | -0.172 | -0.175 | -0.185 | -0.098 | -0.094 | -0.091 | -0.087 |
| Akbar Town | | | | | | | | |
| CI | -0.069 | -0.158 | -0.082 | -0.234 | -0.140 | -0.108 | 0.058 | 0.223 |
| HI | -0.116 | -0.131 | -0.029 | -0.245 | -0.172 | -0.146 | -0.037 | 0.054 |
| BI | -0.142 | -0.112 | 0.023 | -0.223 | -0.160 | -0.137 | -0.032 | 0.045 |
| 3^rd^ Kurana | | | | | | | | |
| CI | -0.168 | -0.002 | 0.240 | 0.198 | 0.279 | -0.053 | -0.117 | -0.121 |
| HI | -0.120 | 0.209 | 0.096 | 0.090 | 0.401* | -0.003 | -0.026 | -0.144 |
| BI | -0.119 | 0.105 | 0.175 | 0.120 | 0.238 | -0.045 | -0.075 | -0.127 |
| Welikadamulla | | | | | | | | |
| CI | -0.195 | -0.106 | -0.127 | -0.162 | 0.105 | 0.192 | 0.041 | -0.172 |
| HI | -0.172 | -0.125 | -0.184 | -0.215 | 0.296 | 0.389* | -0.016 | -0.232 |
| BI | -0.128 | -0.068 | -0.144 | -0.180 | 0.130 | 0.203 | -0.051 | -0.187 |

* Significantly correlated lagged variables with reported dengue incidences during the study period. A 5% significance level was used to identify significant correlations.

Table A6. Summary of correlations between entomological indices and studied meteorological variables.

| Entomological index | *Ae. albopictus* count | | | | *Ae. aegypti* count | | | |
| --- | --- | --- | --- | --- | --- | --- | --- | --- |
|  | Lag0 | Lag1 | Lag2 | Lag3 | Lag0 | Lag1 | Lag2 | Lag3 |
| **CI** | | | | | | | | |
| **Rainfall** | | | | | | | | |
| Eriyawetiya | -0.010 | -0.061 | -0.054 | -0.093 | 0.153 | -0.178 | -0.260 | -0.262 |
| Akbar Town | 0.082 | 0.067 | 0.058 | -0.228 | -0.265 | -0.131 | -0.003 | 0.304 |
| 3^rd^ Kurana | 0.302 | -0.080 | -0.320 | -0.34* | -0.142 | -0.027 | -0.207 | -0.090 |
| Welikadamulla | -0.127 | -0.271 | -0.005 | -0.151 | -0.005 | -0.064 | -0.016 | -0.038 |
| Mawathahena | -0.068 | 0.289 | 0.260 | 0.004 | - | - | - | - |
| **Rainy days** | | | | | | | | |
| Eriyawetiya | -0.225 | -0.179 | 0.041 | -0.105 | -0.146 | -0.222 | -0.185 | -0.059 |
| Akbar Town | -0.162 | 0.063 | -0.089 | -0.120 | 0.016 | 0.069 | 0.303 | 0.227 |
| 3^rd^ Kurana | -0.235 | -0.37* | -0.286 | -0.082 | -0.34* | 0.051 | -0.188 | 0.246 |
| Welikadamulla | -0.37* | -0.136 | 0.265 | -0.055 | -0.042 | 0.191 | -0.026 | 0.015 |
| Mawathahena | 0.066 | 0.306 | 0.116 | 0.005 | - | - | - | - |
| **Minimum temperature** | | | | | | | | |
| Eriyawetiya | -0.153 | -0.199 | -0.35* | -0.026 | 0.121 | 0.017 | 0.109 | -0.035 |
| Akbar Town | 0.091 | -0.199 | 0.061 | 0.019 | 0.250 | 0.321 | -0.183 | -0.147 |
| 3^rd^ Kurana | -0.206 | -0.085 | 0.012 | 0.304 | 0.041 | -0.104 | 0.111 | 0.134 |
| Welikadamulla | 0.238 | 0.131 | 0.193 | 0.337* | 0.242 | 0.065 | 0.048 | -0.185 |
| Mawathahena | 0.044 | -0.258 | 0.139 | -0.113 | - | - | - | - |
| **Maximum temperature** | | | | | | | | |
| Eriyawetiya | -0.066 | 0.227 | 0.007 | 0.298 | -0.36* | -0.180 | 0.039 | -0.066 |
| Akbar Town | 0.049 | 0.076 | -0.139 | 0.133 | 0.185 | -0.155 | -0.015 | -0.274 |
| 3^rd^ Kurana | 0.047 | 0.255 | 0.401* | 0.136 | -0.301 | -0.178 | -0.045 | 0.051 |
| Welikadamulla | 0.272 | 0.189 | -0.056 | -0.110 | 0.077 | -0.175 | -0.169 | 0.057 |
| Mawathahena | 0.109 | -0.223 | 0.032 | -0.135 | - | - | - | - |
| **Minimum RH** | | | | | | | | |
| Eriyawetiya | -0.217 | -0.329 | -0.233 | -0.082 | 0.060 | -0.055 | -0.108 | -0.013 |
| Akbar Town | 0.061 | -0.185 | -0.013 | -0.176 | 0.084 | 0.261 | 0.047 | 0.201 |
| 3^rd^ Kurana | -0.131 | -0.39* | -0.328 | -0.005 | -0.39* | 0.059 | -0.112 | 0.210 |
| Welikadamulla | -0.262 | -0.061 | 0.160 | 0.216 | 0.035 | 0.237 | -0.077 | -0.082 |
| Mawathahena | 0.027 | 0.187 | -0.155 | 0.055 | - | - | - | - |
| **Maximum RH** | | | | | | | | |
| Eriyawetiya | -0.042 | -0.208 | -0.216 | 0.045 | -0.137 | -0.117 | -0.095 | -0.033 |
| Akbar Town | 0.035 | 0.005 | -0.137 | -0.269 | 0.224 | -0.032 | 0.176 | 0.199 |
| 3^rd^ Kurana | 0.050 | -0.266 | -0.52* | -0.004 | -0.35* | -0.132 | -0.173 | 0.378* |
| Welikadamulla | -0.267 | -0.162 | 0.143 | 0.062 | 0.028 | 0.097 | -0.018 | -0.003 |
| Mawathahena | 0.204 | -0.281 | -0.227 | -0.031 | - | - | - | - |
| **Averaged wind speed** | | | | | | | | |
| Eriyawetiya | -0.058 | 0.092 | 0.010 | -0.107 | 0.047 | 0.198 | 0.315 | -0.125 |
| Akbar Town | -0.47* | -0.162 | 0.041 | -0.158 | 0.161 | -0.148 | -0.151 | -0.007 |
| 3^rd^ Kurana | -0.239 | 0.155 | 0.456* | 0.335* | 0.214 | 0.350* | 0.283 | -0.021 |
| Welikadamulla | -0.116 | 0.012 | -0.000 | -0.260 | -0.111 | 0.109 | 0.188 | -0.095 |
| Mawathahena | -0.164 | -0.34* | -0.008 | -0.109 | - | - | - | - |
|  |  |  |  |  |  |  |  |  |
| **HI** | | | | | | | | |
| **Rainfall** | | | | | | | | |
| Eriyawetiya | 0.390* | -0.013 | -0.212 | -0.33* | 0.460* | 0.086 | 0.013 | -0.244 |
| Akbar Town | 0.370* | 0.030 | 0.091 | -0.261 | -0.137 | 0.129 | 0.038 | 0.251 |
| 3^rd^ Kurana | 0.367* | 0.008 | -0.257 | -0.42* | 0.048 | -0.063 | -0.211 | -0.114 |
| Welikadamulla | 0.248 | -0.069 | -0.090 | -0.215 | -0.221 | -0.196 | -0.149 | -0.259 |
| Mawathahena | 0.411* | 0.368* | -0.006 | -0.287 | - | - | - | - |
| **Rainy days** | | | | | | | | |
| Eriyawetiya | -0.035 | -0.45* | -0.127 | -0.207 | 0.291 | -0.117 | -0.058 | -0.34* |
| Akbar Town | -0.109 | -0.265 | -0.265 | -0.124 | 0.190 | 0.039 | 0.182 | 0.097 |
| 3^rd^ Kurana | -0.152 | -0.53* | -0.305 | -0.207 | -0.319 | -0.102 | -0.211 | 0.208 |
| Welikadamulla | -0.046 | -0.239 | -0.063 | -0.058 | 0.166 | 0.145 | -0.37* | -0.004 |
| Mawathahena | +0.098 | -0.024 | -0.40* | -0.174 | - | - | - | - |
| **Minimum temperature** | | | | | | | | |
| Eriyawetiya | -0.213 | -0.108 | -0.246 | 0.197 | -0.035 | 0.031 | 0.014 | 0.230 |
| Akbar Town | -0.053 | -0.269 | -0.044 | 0.125 | 0.080 | 0.139 | -0.241 | -0.135 |
| 3^rd^ Kurana | -0.192 | -0.193 | -0.062 | 0.242 | 0.091 | -0.004 | 0.045 | 0.050 |
| Welikadamulla | 0.096 | -0.110 | 0.117 | 0.051 | 0.239 | -0.026 | 0.174 | -0.229 |
| Mawathahena | 0.024 | -0.283 | 0.066 | -0.027 | - | - | - | - |
| **Maximum temperature** | | | | | | | | |
| Eriyawetiya | -0.262 | 0.260 | 0.119 | 0.336* | -0.252 | -0.178 | -0.004 | -0.019 |
| Akbar Town | 0.016 | 0.195 | 0.076 | 0.262 | 0.084 | -0.202 | -0.048 | -0.068 |
| 3^rd^ Kurana | -0.064 | 0.349* | 0.365* | 0.349* | 0.087 | -0.141 | 0.025 | 0.070 |
| Welikadamulla | -0.175 | 0.068 | 0.121 | 0.003 | 0.015 | -0.38* | 0.007 | 0.032 |
| Mawathahena | -0.234 | -0.184 | 0.226 | 0.069 | - | - | - | - |
| **Minimum RH** | | | | | | | | |
| Eriyawetiya | -0.024 | -0.48* | -0.36* | -0.139 | 0.246 | -0.004 | -0.025 | -0.037 |
| Akbar Town | -0.009 | -0.40* | -0.36* | -0.170 | 0.129 | 0.264 | -0.053 | 0.017 |
| 3^rd^ Kurana | -0.053 | -0.56* | -0.323 | -0.203 | -0.232 | -0.011 | -0.162 | 0.117 |
| Welikadamulla | 0.058 | -0.194 | -0.136 | -0.031 | 0.221 | 0.261 | -0.205 | -0.129 |
| Mawathahena | 0.164 | -0.118 | -0.297 | -0.126 | - | - | - | - |
| **Maximum RH** | | | | | | | | |
| Eriyawetiya | 0.074 | -0.44* | -0.38* | -0.098 | 0.124 | -0.100 | -0.006 | -0.142 |
| Akbar Town | 0.009 | -0.228 | -0.39* | -0.242 | 0.300 | 0.023 | 0.112 | 0.017 |
| 3^rd^ Kurana | 0.069 | -0.38* | -0.38* | -0.178 | -0.35* | -0.186 | -0.176 | 0.264 |
| Welikadamulla | -0.101 | -0.135 | -0.114 | -0.124 | 0.074 | 0.130 | -0.327 | -0.099 |
| Mawathahena | 0.069 | -0.062 | -0.328 | -0.094 | - | - | - | - |
| **Averaged wind speed** | | | | | | | | |
| Eriyawetiya | -0.302 | -0.058 | 0.161 | 0.018 | -0.085 | -0.158 | 0.339* | 0.145 |
| Akbar Town | -0.55* | -0.068 | 0.094 | -0.081 | 0.135 | -0.248 | -0.259 | 0.015 |
| 3^rd^ Kurana | -0.277 | 0.231 | 0.331* | 0.338* | 0.229 | 0.427* | 0.384* | 0.034 |
| Welikadamulla | -0.312 | 0.003 | 0.044 | -0.162 | -0.257 | 0.022 | 0.255 | 0.106 |
| Mawathahena | -0.251 | -0.020 | 0.210 | 0.126 | - | - | - | - |
|  |  |  |  |  |  |  |  |  |
| **BI** | | | | | | | | |
| **Rainfall** | | | | | | | | |
| Eriyawetiya | 0.408* | -0.025 | -0.235 | -0.326 | 0.460* | 0.086 | 0.013 | -0.244 |
| Akbar Town | 0.393* | 0.118 | -0.067 | -0.275 | -0.105 | 0.158 | 0.031 | 0.210 |
| 3^rd^ Kurana | 0.414* | -0.017 | -0.302 | -0.36* | -0.033 | -0.116 | -0.239 | -0.098 |
| Welikadamulla | 0.212 | -0.124 | -0.049 | -0.192 | 0.212 | 0.225 | -0.076 | -0.225 |
| Mawathahena | 0.349* | 0.344* | 0.089 | -0.243 | - | - | - | - |
| **Rainy days** | | | | | | | | |
| Eriyawetiya | -0.015 | -0.46* | -0.134 | -0.176 | 0.291 | -0.117 | -0.058 | -0.34* |
| Akbar Town | -0.001 | -0.224 | -0.277 | -0.128 | 0.198 | 0.078 | 0.153 | 0.082 |
| 3^rd^ Kurana | -0.192 | -0.48* | -0.35* | -0.156 | -0.37* | -0.033 | -0.214 | 0.232 |
| Welikadamulla | -0.080 | -0.283 | 0.033 | -0.023 | 0.200 | 0.099 | -0.239 | -0.026 |
| Mawathahena | 0.064 | 0.015 | -0.306 | -0.165 | - | - | - | - |
| **Minimum temperature** | | | | | | | | |
| Eriyawetiya | -0.264 | -0.079 | -0.274 | 0.232 | -0.035 | 0.031 | 0.014 | 0.230 |
| Akbar Town | -0.145 | -0.247 | -0.115 | 0.028 | 0.059 | 0.152 | -0.203 | -0.115 |
| 3^rd^ Kurana | -0.211 | -0.180 | -0.072 | 0.260 | 0.040 | -0.130 | 0.084 | 0.186 |
| Welikadamulla | 0.099 | -0.086 | 0.174 | 0.224 | 0.137 | -0.029 | 0.089 | -0.212 |
| Mawathahena | 0.024 | -0.309 | 0.063 | 0.036 | - | - | - | - |
| **Maximum temperature** | | | | | | | | |
| Eriyawetiya | -0.293 | 0.266 | 0.057 | 0.343* | -0.252 | -0.178 | -0.004 | -0.019 |
| Akbar Town | 0.042 | 0.161 | 0.068 | 0.324 | 0.113 | -0.267 | -0.004 | -0.059 |
| 3^rd^ Kurana | -0.023 | 0.344* | 0.443* | 0.296 | 0.212 | -0.089 | 0.051 | -0.001 |
| Welikadamulla | -0.043 | 0.193 | 0.022 | 0.060 | 0.068 | -0.35* | 0.037 | 0.008 |
| Mawathahena | -0.237 | -0.121 | 0.154 | 0.065 | - | - | - | - |
| **Minimum RH** | | | | | | | | |
| Eriyawetiya | 0.005 | -0.50* | -0.35* | -0.095 | 0.246 | -0.004 | -0.025 | -0.037 |
| Akbar Town | 0.172 | -0.331 | -0.39* | -0.281 | 0.119 | 0.293 | -0.046 | 0.015 |
| 3^rd^ Kurana | -0.053 | -0.56* | -0.323 | -0.203 | -0.232 | -0.011 | -0.162 | 0.117 |
| Welikadamulla | -0.049 | -0.253 | -0.003 | 0.063 | 0.216 | 0.208 | -0.196 | -0.168 |
| Mawathahena | 0.169 | -0.142 | -0.208 | -0.093 | - | - | - | - |
| **Maximum RH** | | | | | | | | |
| Eriyawetiya | 0.120 | -0.45* | -0.41* | -0.047 | 0.124 | -0.100 | -0.006 | -0.142 |
| Akbar Town | 0.146 | -0.127 | -0.39* | -0.309 | 0.299 | 0.021 | 0.093 | 0.059 |
| 3^rd^ Kurana | 0.057 | -0.36* | -0.43* | -0.104 | -0.39* | -0.181 | -0.179 | -0.39* |
| Welikadamulla | -0.196 | -0.238 | -0.007 | 0.0002 | 0.205 | 0.107 | -0.283 | -0.183 |
| Mawathahena | 0.111 | -0.034 | -0.277 | -0.053 | - | - | - | - |
| **Averaged wind speed** | | | | | | | | |
| Eriyawetiya | -0.302 | -0.026 | 0.190 | 0.083 | -0.085 | -0.158 | 0.339* | 0.145 |
| Akbar Town | -0.47* | -0.239 | -0.010 | 0.013 | 0.078 | -0.228 | -0.217 | 0.016 |
| 3^rd^ Kurana | -0.265 | 0.209 | 0.438* | 0.330 | 0.184 | 0.360* | 0.348* | 0.051 |
| Welikadamulla | -0.267 | 0.140 | -0.027 | -0.155 | -0.35* | -0.138 | 0.167 | 0.095 |
| Mawathahena | -0.272 | -0.047 | 0.158 | 0.065 | - | - | - | - |

* Significantly correlated lagged variables with each studied climatic factor during the study period. A 5% significance level was used to identify significant correlations.

Table A7. Summary of correlations of field-caught dengue vector mosquitoes with reported patient cases in the study areas.

| Category | Lag0 | Lag1 | Lag2 | Lag3 |
| --- | --- | --- | --- | --- |
| Eriyawetiya | | | | |
| Total mosquito count | -0.027 | 0.058 | -0.009 | 0.043 |
| Count of *Ae. aegypti* | 0.395* | 0.549* | 0.090 | 0.075 |
| Count of *Ae. albopictus* | -0.042 | 0.038 | -0.013 | 0.040 |
| Akbar Town | | | | |
| Total mosquito count | -0.216 | -0.090 | 0.002 | -0.067 |
| Count of *Ae. aegypti* | -0.054 | 0.220 | 0.606* | -0.064 |
| Count of *Ae. albopictus* | -0.218 | -0.119 | -0.068 | -0.062 |
| 3^rd^ Kurana | | | | |
| Total mosquito count | -0.013 | -0.062 | 0.267 | 0.024 |
| Count of *Ae. aegypti* | 0.276 | -0.024 | 0.002 | 0.257 |
| Count of *Ae. albopictus* | -0.039 | -0.061 | 0.271 | 0.001 |
|  |  |  |  |  |
| Welikadamulla | | | | |
| Total mosquito count | -0.099 | -0.122 | -0.271 | -0.271 |
| Count of *Ae. aegypti* | 0.027 | 0.012 | -0.078 | -0.067 |
| Count of *Ae. albopictus* | -0.105 | -0.127 | -0.271 | -0.272 |
| Mawathahena (Control area) | | | | |
| Total mosquito count | - | - | - | - |
| Count of *Ae. aegypti* | - | - | - | - |
| Count of *Ae. albopictus* | - | - | - | - |

* Significantly correlated lagged variables with reported dengue incidences during the study period. A 5% significance level was used to identify significant correlations. Since no dengue cases was reported from the control area, no correlation was observed.

Table A8. Summary of correlations of field-caught dengue vector mosquitoes with studied climatic variables in the study areas.

| Category | Total mosquito count | | | | Count of *Ae. aegypti* | | | | Count of *Ae. albopictus* | | | |
| --- | --- | --- | --- | --- | --- | --- | --- | --- | --- | --- | --- | --- |
|  | Lag0 | Lag1 | Lag2 | Lag3 | Lag0 | Lag1 | Lag2 | Lag3 | Lag0 | Lag1 | Lag2 | Lag3 |
| Eriyawetiya | | | | | | | | | | | | |
| Rainfall | 0.298 | -0.178 | -0.271 | -0.344* | 0.107 | -0.196 | -0.285 | -0.018 | 0.298 | -0.172 | -0.263 | -0.348* |
| Number of rainy days | 0.236 | -0.188 | -0.366* | -0.452* | 0.201 | -0.237 | -0.318 | -0.030 | 0.232 | -0.181 | -0.358* | -0.456* |
| Minimum temperature | 0.006 | -0.061 | -0.074 | 0.081 | -0.049 | -0.045 | 0.032 | 0.050 | 0.008 | -0.060 | -0.076 | 0.080 |
| Maximum temperature | -0.168 | -0.145 | -0.098 | -0.017 | -0.147 | -0.066 | 0.139 | 0.001 | -0.165 | -0.144 | -0.104 | -0.017 |
| Minimum RH | 0.243 | -0.028 | -0.181 | -0.200 | 0.127 | -0.108 | -0.269 | -0.015 | 0.241 | -0.024 | -0.173 | -0.202 |
| Maximum RH | 0.283 | -0.102 | -0.292 | -0.295 | 0.152 | -0.154 | -0.362* | -0.094 | 0.280 | -0.097 | -0.282 | -0.295 |
| Averaged wind speed | -0.085 | 0.201 | 0.363 | -0.028* | 0.073 | 0.153 | 0.399* | 0.215 | -0.089 | 0.198 | 0.353* | -0.037 |
| Akbar Town | | | | | | | | | | | | |
| Rainfall | 0.114 | 0.162 | -0.009 | -0.331* | 0.241 | 0.028 | -0.274 | -0.186 | 0.091 | 0.165 | 0.022 | -0.323* |
| Number of rainy days | 0.032 | 0.128 | -0.134 | -0.319 | 0.182 | 0.174 | -0.233 | -0.125 | 0.012 | 0.113 | -0.112 | -0.318 |
| Minimum temperature | 0.111 | -0.027 | 0.109 | 0.186 | 0.111 | -0.005 | 0.041 | -0.004 | 0.102 | -0.028 | 0.109 | 0.194 |
| Maximum temperature | 0.238 | 0.164 | 0.019 | 0.129 | 0.021 | -0.234 | -0.125 | -0.151 | 0.245 | 0.198 | 0.034 | 0.152 |
| Minimum RH | 0.108 | 0.075 | 0.007 | -0.144 | 0.186 | 0.200 | -0.158 | -0.070 | 0.091 | 0.054 | 0.025 | -0.141 |
| Maximum RH | 0.171 | 0.212 | -0.041 | -0.285 | 0.171 | 0.127 | 0.344* | -0.231 | 0.158 | 0.206 | -0.003 | -0.269 |
| Averaged wind speed | -0.418* | 0.141 | -0.017 | 0.019 | -0.168 | 0.169 | 0.229 | 0.173 | -0.415* | 0.127 | -0.044 | 0.000 |
| 3^rd^ Kurana | | | | | | | | | | | | |
| Rainfall | 0.418 | -0.113 | -0.395* | -0.231 | -0.173 | -0.094 | -0.185 | -0.137 | 0.439* | -0.106 | -0.383* | -0.221 |
| Number of rainy days | 0.314 | -0.128 | -0.409* | -0.321 | -0.218 | -0.219 | -0.188 | -0.069 | 0.338* | -0.110 | -0.397* | -0.319 |
| Minimum temperature | -0.169 | -0.158 | -0.290 | -0.176 | -0.301 | -0.208 | -0.045 | 0.037 | -0.144 | -0.141 | -0.290 | -0.181 |
| Maximum temperature | -0.268 | -0.020 | 0.352* | 0.413* | 0.092 | 0.122 | 0.274 | 0.262 | -0.280 | -0.031 | 0.331 | 0.394* |
| Minimum RH | 0.254 | -0.125 | -0.502* | -0.422* | -0.308 | -0.198 | -0.217 | -0.130 | 0.285 | -0.108 | -0.488* | -0.416* |
| Maximum RH | 0.461* | 0.052* | -0.372* | -0.270 | -0.067 | 0.069 | -0.209 | -0.054 | 0.473* | 0.046 | -0.357* | -0.269 |
| Averaged wind speed | -0.227 | 0.130 | 0.503* | 0.266 | 0.129 | -0.021 | 0.086 | 0.008 | -0.241 | 0.134 | 0.502* | 0.268 |
|  |  |  |  |  |  |  |  |  |  |  |  |  |
| Welikadamulla | | | | | | | | | | | | |
| Rainfall | 0.227 | -0.016 | -0.068 | 0.112 | 0.098 | 0.164 | 0.131 | 0.102 | 0.223 | -0.035 | -0.085 | 0.104 |
| Number of rainy days | 0.191 | 0.070 | -0.109 | -0.020 | 0.163 | 0.177 | 0.096 | -0.060 | 0.179 | 0.052 | -0.124 | -0.014 |
| Minimum temperature | 0.068 | 0.056 | 0.217 | 0.145 | -0.033 | -0.024 | -0.088 | -0.141 | 0.074 | 0.061 | 0.235 | 0.166 |
| Maximum temperature | 0.151 | 0.174 | 0.271 | 0.178 | -0.186 | -0.084 | -0.057 | 0.155 | 0.178 | 0.190 | 0.287 | 0.166 |
| Minimum RH | 0.194 | 0.050 | 0.005 | 0.072 | 0.189 | 0.179 | 0.118 | -0.116 | 0.179 | 0.032 | -0.008 | 0.088 |
| Maximum RH | 0.216 | 0.155 | 0.035 | 0.017 | 0.269 | 0.314 | 0.238 | 0.010 | 0.193 | 0.124 | 0.009 | 0.017 |
| Averaged wind speed | -0.277 | 0.050 | -0.083 | -0.174 | -0.117 | -0.182 | -0.375* | 0.127 | -0.273 | 0.072 | -0.043 | -0.195 |
| Mawathahena (Control area) | | | | | | | | | | | | |
| Rainfall | 0.464* | -0.025 | -0.281 | -0.439* | 0.090 | -0.024 | -0.128 | -0.103 | 0.460* | -0.025 | -0.277 | -0.435* |
| Number of rainy days | 0.463* | -0.014 | -0.138 | -0.407* | 0.096 | -0.080 | -0.238 | -0.156 | 0.459* | -0.012 | -0.131 | -0.402* |
| Minimum temperature | 0.252 | 0.193 | 0.100 | 0.001 | -0.118 | -0.180 | -0.358* | -0.233 | 0.255 | 0.197 | 0.110 | 0.008 |
| Maximum temperature | -0.245 | -0.320 | -0.201 | 0.038 | -0.185 | -0.047 | 0.038 | 0.276 | -0.239 | -0.318 | -0.202 | 0.030 |
| Minimum RH | 0.388* | 0.183 | -0.069 | -0.276 | 0.147 | -0.065 | -0.267 | -0.261 | 0.383* | 0.184 | -0.061 | -0.268 |
| Maximum RH | 0.247 | -0.108 | -0.398* | -0.398* | 0.287 | 0.127 | -0.082 | -0.153 | 0.239 | -0.111 | -0.395* | -0.393* |
| Averaged wind speed | -0.011 | 0.189 | 0.451* | 0.361* | -0.399* | -0.115 | -0.051 | 0.081 | 0.000 | 0.191 | 0.452* | 0.358* |

* Significantly correlated lagged variables with meteorological variables during the study period. A 5% significance level was used to identify significant correlations.

Table A9. Summary of dengue vector mosquito larvae and egg pools collected from study areas.

| Study area | Larvae pools | Egg pools | Total number of pools |
| --- | --- | --- | --- |
| Eriyawetiya | 26 | 8 | 34 |
| Akbar Town | 37 | 8 | 45 |
| 3^rd^ Kurana | 23 | 9 | 32 |
| Welikadamulla | 33 | 12 | 45 |
| Mawathahena (Control) | 32 | 4 | 36 |
